# Nitrate and the Origin of Saliva Influence Composition and Short Chain Fatty Acid Production of Oral Microcosms

**DOI:** 10.1007/s00248-016-0775-z

**Published:** 2016-05-07

**Authors:** Jessica E. Koopman, Mark J. Buijs, Bernd W. Brandt, Bart J. F. Keijser, Wim Crielaard, Egija Zaura

**Affiliations:** Department of Preventive Dentistry, Academic Centre for Dentistry Amsterdam, University of Amsterdam and VU University Amsterdam, Gustav Mahlerlaan 3004, 1081 LA Amsterdam, The Netherlands; Research Group Microbiology and Systems Biology, TNO Earth, Life and Social Sciences, Utrechtseweg 48, 3704 HE Zeist, The Netherlands

**Keywords:** Oral microbiome, Nitrate reduction, Short chain fatty acids, *Veillonella*, *Neisseria*

## Abstract

Nitrate is emerging as a possible health benefactor. Especially the microbial conversion of nitrate to nitrite in the oral cavity and the subsequent conversion to nitric oxide in the stomach are of interest in this regard. Yet, how nitrate influences the composition and biochemistry of the oral ecosystem is not fully understood. To investigate the effect of nitrate on oral ecology, we performed a 4-week experiment using the multiplaque artificial mouth (MAM) biofilm model. This model was inoculated with stimulated saliva of two healthy donors. Half of the microcosms (*n* = 4) received a constant supply of nitrate, while the other half functioned as control (*n* = 4). Additionally, all microcosms received a nitrate and sucrose pulse, each week, on separate days to measure nitrate reduction and acid formation. The bacterial composition of the microcosms was determined by 16S rDNA sequencing. The origin of the saliva (i.e., donor) showed to be the strongest determinant for the development of the microcosms. The supplementation of nitrate was related to a relatively high abundance of *Neisseria* in the microcosms of both donors, while *Veillonella* was highly abundant in the nitrate-supplemented microcosms of only one of the donors. The lactate concentration after sucrose addition was similarly high in all microcosms, irrespective of treatment or donor, while the concentration of butyrate was lower after nitrate addition in the nitrate-receiving microcosms. In conclusion, nitrate influences the composition and biochemistry of oral microcosms, although the result is strongly dependent on the inoculum.

**Electronic supplementary material:**

The online version of this article (doi:10.1007/s00248-016-0775-z) contains supplementary material, which is available to authorized users.

## Introduction

For many years, the role of nitrate in the human body has been under debate and usually not in favor of nitrate [1–5]. Nowadays, the view on nitrate ingestion has started to change drastically. Especially the bacterial reduction of nitrate to nitrite in the oral cavity is of interest, since the formed nitrite can be converted to the potential health-beneficial nitric oxide [6–8].

The ability to reduce nitrate is widespread in prokaryotes and fungi. Recently, nitrate-reducing capacity was discovered to be present in certain human tissues, although this capacity is very low compared to the nitrate reduction performed by bacteria in the human oral cavity [9–12]. Many bacteria in the oral cavity possess genes that are involved in the reduction of nitrate [13, 14]. As a good example of commensalism, the nitrate that is ingested by the host by eating for instance leafy vegetables is concentrated in the saliva (around 10× compared to plasma) and used by nitrate-reducing bacteria in the oral cavity [15–18].

There are three groups of bacterial nitrate reductases, namely the periplasmic dissimilatory reductases (Nap), membrane-bound respiratory reductases (Nar), or the cytoplasmic assimilatory reductases (Nas). Notwithstanding their differences in function and cell location, all bacterial nitrate reductases have a conserved molybdenum binding site [19–22]. Some years ago, it was discovered that the concentration of molybdenum in soil is negatively correlated to caries prevalence in humans [23, 24], although it remained unclear how molybdenum contributed to a lower caries prevalence. Moreover, at that time, it was not recognized that molybdenum was essential to nitrate reductase [25].

Now, nitrate and especially the microbial ability to reduce nitrate to nitrite in the oral cavity is thought to have an anti-caries effect [13, 26, 27]. Several suggestions have been made to explain the mechanism of this effect. For example, the fatty acids associated with caries formation are used as a carbon source in the nitrate reduction pathway, ammonium is produced through the nitrate reduction pathway, elevating the pH in the oral cavity, or the formation of nitric oxide in the vicinity of acid-producing bacteria has a bactericidal effect [26–28].

In contrast to the possible anti-caries activity of nitrate, elevated levels of nitrate and nitrite are associated with periodontal disease [29]. However, this elevation is thought to be a response of the immune system against infection or stress [30].

So far, a wealth of information has been gathered on the different nitrate reduction pathways in bacteria, and the role of nitrate in human physiology has been a topic of research for quite some time. However, there is no comprehensive knowledge on the effect of nitrate on the composition of the oral bacterial population and their metabolism.

We performed an in vitro study using saliva of two healthy donors to inoculate the multiplaque artificial mouth (MAM) system [31, 32]. We determined the bacterial composition of the microcosms as well as the formation of different short chain fatty acids and the nitrate-reducing ability in the model system and assays. The main advantage of this in vitro study is that there are no host factors involved, which allows us to focus solely on the microbial function and composition.

Hence, the aim of our study was to elucidate the effect of a continuous supply of nitrate on the nitrate-reducing ability and acid production of the oral microbiome, in addition to the composition of the microbiome itself.

## Materials and Methods

### Inoculation of the Artificial Mouth

The eight-station MAM biofilm model (Fig. S1) was designed and developed by Dr. Christopher Sissons and Dr. Lisa Wong [31, 32]. To grow the microcosms, the MAM was inoculated with stimulated saliva. The saliva was obtained from two donors (20–30 years), one male and one female, who were in good systemic health and had not used antibiotics 6 months prior to the experiment. The subjects were examined by a dentist to ascertain good oral health status, e.g., no active caries, gingivitis, or periodontitis. The donors were asked to refrain from oral hygiene 12 h prior to collection. The stimulated saliva was collected by chewing on gum base (Wrigley, Chicago, IL, USA) while expectorating the saliva in a sterile container until 10 ml was obtained. Directly after collection, the saliva was vortexed thoroughly for 30 s and used to inoculate ∅25 mm Thermanox coverslips (Nunc Inc. Naperville, IL, USA). The coverslips (*n* = 8) were inoculated with 1 ml saliva each, four per donor, and aerobically incubated for 1 h at 35 °C before placement in the MAM.

### Experimental Conditions

The microcosms were grown for 31 days. Throughout the experiment, the temperature of the system was kept at 35 °C and the MAM was constantly flushed with nitrogen gas containing 5 % CO_2_, although the system was not regarded as strictly anaerobic due to the opening and closing of the ports during sampling.

### Continuous Supply of Medium

All stations received a constant supply (0.06 ml/min) of defined mucin medium (DMM) [33], to which a trace element solution DSMZ SL-4 (Table S1, DSMZ GmbH, Braunschweig, Germany) was added. The pH of the DMM was set at 6.8 using NaOH. The medium was supplied through a 0.25-mm bore Marprene Manifold pump tube (Watson-Marlow Limited, Falmouth, England), lead through a 205CA 16-channel pump head connected to a 505DUpump (Watson-Marlow).

To the DMM reservoirs of four of the stations (1, 3, 5, and 7), nitrate was added to a final concentration of 1 mM from a 1-M nitrate solution (prepared by dissolving 6.77 g KNO_3_ and 2.80 g NaNO_3_ in 100 ml Milli-Q water (Millipore, Billeria, MA, USA)). The two different treatments that the stations received will be referred to as control or nitrate (Fig. S2).

### Daily Supply of Sucrose

The microcosms received daily doses of sucrose to create a diurnal cycle mimicking a resting and fermentation period. All stations were connected to a sucrose reservoir and received eight pulses of 6 min 10 % *w*/*v* sucrose (Merck KGaA, Darmstadt, Germany) at 2 h intervals daily. The pulses (0.5 ml/min) were supplied automatically through a 1.3-mm bore pump tube (Ismatec, Wertheim, Germany) using a 503U pump with a 308MC pump head (Watson-Marlow), controlled by LabView v7.0 (National Instruments Corporation, Austin, TX, USA). The first sucrose pulse commenced at 17:00 hours and the last pulse at 07:00 hours.

### Weekly Supply of Sucrose and Nitrate

In addition to the continuous and daily supply of nitrate and sucrose, respectively, the two compounds were added to the microcosms weekly to observe the nitrate reduction and sucrose metabolic activity of the all the microcosms.

On days 8, 15, 22, and 29, a manual 6 min 10 % *w*/*v* sucrose pulse was supplied to all the microcosms. Nitrate was manually added to all microcosms on days 10, 17, 24, and 31. To provide this manual 6-min nitrate pulse, all stations were connected to a reservoir containing a 5-mM nitrate solution. The nitrate solution was supplied through a 0.63-mm bore Marprene Manifold pump tubing (Watson-Marlow Limited), using a 505Du pump with a 308MC pump head, rotating at 10 rpm (0.3 ml/min).

### Sampling

Biomass was harvested from the microcosms using a pipette and a sterile 1-ml filtertip (Biosphere, Sarstedt, Nümbrecht, Germany). The sample was taken throughout the depth of the biofilm.

To observe nitrate reduction and sucrose metabolic activity in the microcosms, samples were collected before (*t* = 0 min) the nitrate or sucrose pulse, directly after the pulse (*t* = 6 min), and 1 h after the pulse (*t* = 60 min).

In addition, to confirm the nitrate and short chain fatty acid concentration measurements within the microcosms, samples of the microcosms were collected to perform assays measuring nitrate reduction and sucrose metabolic capacity. These samples were taken on days 9, 16, 23, and 30, when the biofilms did not receive a pulse.

Samples to be used for DNA isolation were taken twice a week on the days the microcosms received a manual pulse of either nitrate or sucrose at *t* = 0 min. The *t* = 0 min samples were always taken around 12:00 hours.

The samples to be used for the assays and the acid and nitrate analysis were suspended in Milli-Q water and samples to be used for DNA isolation were suspended in PBS. The samples were placed on ice directly and later stored at −80 °C until further use.

### Nitrate Reduction Assay

The biomass used in the nitrate reduction assay was suspended, in duplicate, in 100 μl 0.1 M PO_4_^3−^ buffer and spun for 1 min at 16,000×*g*. The pellet was resuspended in a reaction buffer containing 10 mM PO_4_^3−^, 0.11 mM pyruvate, and 1 mM KNO_3_/NaNO_3_ and incubated for 1 h at 37 °C. One of the duplicate samples was incubated aerobically, the other anaerobically. The reaction vials were stored at −80 °C until later analysis.

### Nitrate and Nitrite Analysis

The concentration of nitrate and nitrite that was reduced and formed in the assays (“Nitrate Reduction Assay” section) and in the microcosms after the manual nitrate pulse was determined using capillary electrophoresis. First, the samples were centrifuged at 13,000×*g* for 15 min at 4 °C. The supernatants were transferred to tubes containing a 0.22-μm microspin filter (Ultrafree-MC, Millipore, Bedford, MA, USA) and centrifuged at 12,000×*g* for 5 min at 4 °C. Filtered supernatants were stored at −80 °C.

The capillary electrophoresis was performed on a Beckman P/ACE™ MDQ (Beckman Coulter, Brea, CA, USA) system at 25 °C with UV detection at 214 nm, capillary length of 50 cm, and separation at 25 kV in reverse mode. Run buffers were derived from the CEofix™ Anions 2 kit (Analis, Suarlée, Belgium). Potassium bromate was used as the internal standard in all samples.

### Enzymatic Ammonium Assay

The concentration of ammonium that was produced during the nitrate reduction assay was measured using an enzymatic ammonium assay as described by Hoogenkamp and ten Cate [34].

### Sucrose Metabolism Assay

To perform a sucrose metabolism assay outside of the MAM system, biomass collected from the microcosms was suspended in 37 °C sterile saline (Milli-Q water containing 0.9 % NaCl). The samples were spun for 1 min at 16,000×*g*. The supernatant was discarded and the pellet was resuspended in saline (37 °C). The pH of the samples was measured. After briefly spinning these samples, 50 μl was transferred to a clean tube and placed on ice before storage at −80 °C. These samples were used as baseline measurements for short chain fatty acid analysis.

To the remaining sample, 16 μl 10 % (*w*/*v*) sucrose was added and the pH was measured every minute for a total of 10 min. The samples were then placed on ice before storage at −80 °C. These samples were later used for the analysis of the concentration of phosphate and the short chain fatty acids formate, succinate, lactate, propionate, acetate, and butyrate.

### Short Chain Fatty Acid and Phosphate Analysis

The concentration of short chain fatty acids and phosphate formed in the assays (“Sucrose Metabolism Assay” section) and the concentration of short chain fatty acids formed in the microcosms after the manual sucrose pulse were determined using capillary electrophoresis.

To release acids and phosphate, the samples were heated at 80 °C for 5 min and cooled on ice. Subsequently, the samples were centrifuged at 13,000×*g* for 15 min at 4 °C. The supernatants were transferred to tubes containing a 0.22-μm microspin filter (Millipore) and centrifuged at 12,000×*g* for 5 min at 4 °C. The filtered supernatants were stored at −80 °C until further processing [35].

Short chain fatty acids and phosphate were determined as their anions by capillary electrophoresis on the Beckman P/ACE™ MDQ system with UV detection at 230 nm and capillary length of 90 cm in reverse mode. Run buffers from the CEofix™ Anions 5 kit (Analis, Suarlée, Belgium) were used. Sodium salts of formate, acetate, propionate, butyrate, succinate, lactate, and phosphate were used to prepare standard solutions in Milli-Q water. Calibration curves were made for each compound separately. As an internal standard, oxalate was included in all samples.

### Determination of Protein Concentration

The protein concentration of the samples was determined to measure the biomass. This was done for all samples, with the exception of the samples used for DNA analyses.

The samples were spun down, the supernatant was discarded, and the pellet was resuspended in 200 μl Milli-Q water. Subsequently, the pellets were homogenized by sonication for 30 s on ice (1 s intervals, Amplitude 40, Vibra-Cell™, Sonics Materials, Inc, Newtown, CT, USA). Duplicate 10 μl aliquots were taken for the analysis of water-soluble protein using the Bradford reagent (Sigma-Aldrich, St. Louis, MO, USA) [36, 37].

### Genomic DNA Extraction

The GeneJET Genomic DNA Purification Kit (Thermo Fisher Scientific, Waltham, MA, USA) with a partially adapted protocol was used for DNA isolation. The biofilm pellet was suspended in 750 μl lysis solution and transferred to a 2.0-ml cryovial containing ∅0.1 mm glass beads (BioSpec Products, Inc., Bartlesville, OK, USA). Beadbeating was done in the MiniBeadBeater (BioSpec Products, Inc.) three times for 2 min. In between the beadbeating steps, the vials were incubated on ice for 5 min. Subsequently, proteinase K was added to the vials and the isolation was continued according to the manufacturer’s protocol (gram-positive bacteria genomic DNA purification protocol).

### 16S rDNA Sequencing

The concentration of DNA was measured using qPCR [38] and normalized to 2 ng per PCR reaction. The V4 region of the 16S rRNA gene was amplified [39] with primers containing the respective Illumina adapters and a unique 8-nt index sequence key [40]. The amplification was performed according to Kozich et al. [40], with the exception of 33 cycles instead of 35. The amount of DNA per sample was quantified using the Quant-iT™ PicoGreen® dsDNA Assay Kit (Thermo Fisher Scientific), pooled equimolarly, and purified using the Illustra™ GFX™ PCR DNA and Gel Band Purification Kit (GE Healthcare, Eindhoven, the Netherlands). The quality and size of the amplicons was analyzed on the 2100 Bioanalyzer (Agilent Technologies, Santa Clara, CA, USA). Paired-end sequencing (200 cycles) of the DNA was conducted on the MiSeq platform (Illumina, San Diego, CA, USA) at TNO (Zeist, the Netherlands). The flow cell was loaded with 6 pmol DNA containing 50 % PhiX. The sequence data have been submitted to the Sequence Read Archive (SRA) under accession number prjna308439.

### Sequencing Data Analysis

As the sequences of a read pair overlap, the paired reads were first merged using USEARCH v8.0.1623 [41, 42] (max number of mismatches in the overlap, 15; max length of the merged reads, 258; min length of the merged reads, 249; max expected error, 0.5; no ambiguous bases were allowed). Before clustering, the sorted reads were checked against the Illumina PhiX RTA reference, using both local and global alignment (USEARCH with -id 0.5 -query_cov 0.5) to exclude the possibility that PhiX reads were included during clustering. The merged sequences were clustered into operational taxonomic units (OTUs), in line with the UPARSE pipeline [43] (with the following adaptations: cluster_otus with -uparse_maxdball 1200, only de novo chimera checking, and usearch_global with -maxaccepts 8 -maxrejects 64 -maxhits 1). The most abundant read of each OTU was assigned a taxonomy using QIIME v1.8.0 [44], the RDP classifier [45] (min confidence 0.8), and the SILVA 119 database [46]. The alignment of the 97 % representative 16S ribosomal DNA (rDNA) sequence set, provided by the QIIME developers, was first trimmed to the V4 region [47], and the alignment was converted to a set of gap-free nonredundant sequences. This set was used to retrain the RDP classifier. The resulting OTU table was randomly subsampled to an equal depth per sample using QIIME (single_rarefaction.py).

In an attempt to identify relevant OTUs on species level, the representative sequence of each OTU was aligned, using MegaBLAST [48, 49], against NCBI’s nucleotide collection (nr/nt), excluding uncultured/environmental sample sequences on the NCBI BLAST web server using default settings. Multiple matches with the same top score for any representative OTU sequence analyzed were not encountered.

### Statistical Analysis

To visualize the position of the microbial profiles per week and treatment relative to each other, a nonmetric multidimensional scaling plot (nmMDS) based on the Bray-Curtis distance was calculated in PAST v3.0 [50]. One-way permutational multivariate analysis of variance (PERMANOVA) was performed in PAST to determine if there was statistical difference between the microcosm composition with regard to treatment. The Shannon diversity index per sample was calculated in PAST as well. Whether there was a significant difference in Shannon diversity index between treatments was tested with the Wilcoxon signed ranks test using SPSS v21 (IBM Corp, Armonk, NY, USA). Linear discriminant analysis effect size (LEfSe) was used to determine which genera and OTUs were significantly differentially abundant between the two treatments [51].

The Wilcoxon signed ranks test was also used to determine whether the concentrations of nitrate, nitrite, ammonium, phosphate, formate, acetate, propionate, butyrate, succinate, and lactate were significantly different between time points (either in the MAM or in the assays) within the same treatment group. The Mann-Whitney test was used to determine whether the concentrations were significantly different at the same time points between the two treatments. The biochemical data, collected during 4 weeks, were analyzed together and not per week. All statistical calculations were performed for each donor separately.

## Results

We have analyzed the effect of nitrate on the microbial composition and biochemistry of oral microcosms. Stimulated saliva from two donors was used to inoculate four microcosms per donor. The data obtained from the microcosms have been analyzed per donor.

### Sequencing Output

The total number of reads after merging, quality filtering, clustering, and mapping was 1,721,028, with an average number of 26,076 reads per sample (SD 4721, min 18,446, max 39,446). The subsampling depth was set at 18,000 reads per sample.

The number of OTUs in the inoculum of donor A was 89, while the average number of OTUs in the microcosms derived from donor A was 48.5 (SD 4.7, min 38, max 58). For donor B, the number of OTUs in the inoculum was 87, while the average number of OTUs in the microcosms derived from donor B was 39 (SD 11.0, min 20, max 61).

### Community Composition

**Fig. 1:**
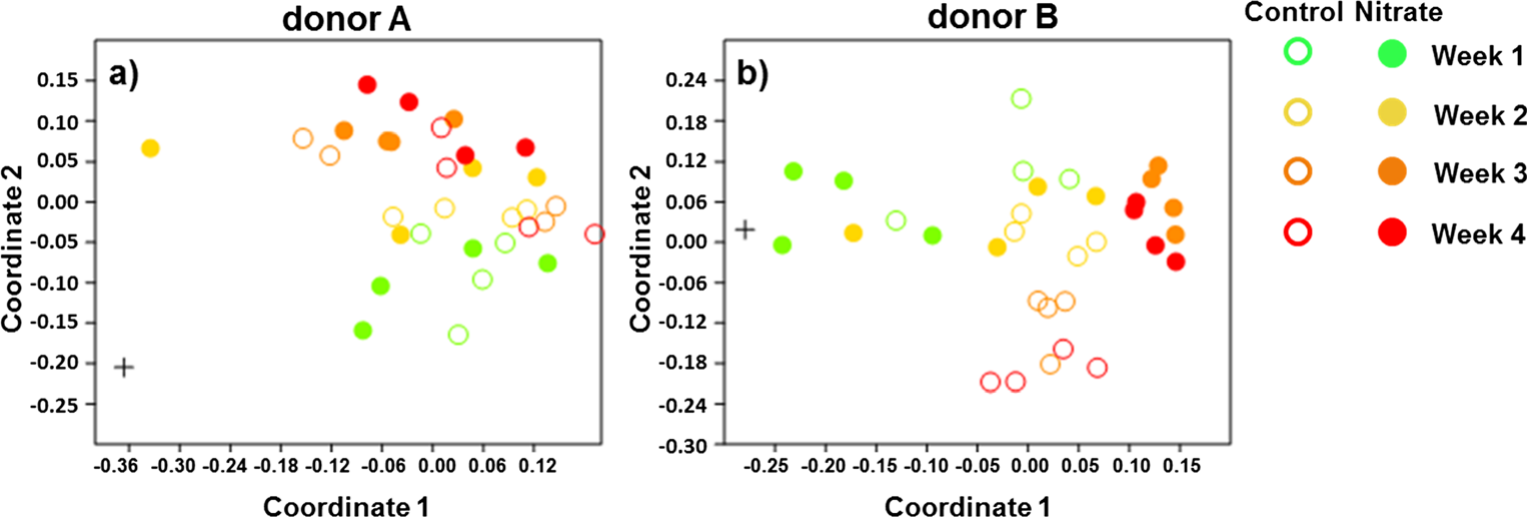
Nonmetric multidimensional scaling plots based on the three-dimensional Bray-Curtis similarity index based on time and treatment. The plots depict the similarities between the microcosms derived from donor A (stress 0.1027) (**a**) and the similarities between the microcosms derived from donor B (stress 0.1340) (**b**). The *plus symbol* represents the inoculum, the *open dots* represent the control microcosms, and the *solid dots* represent the nitrate treatment. The *green dots* represent week 1, the *yellow dots* represent week 2, the *orange dots* represent week 3, and the *red dots* represent week 4

The nonmetric multidimensional scaling plot (nmMDS) revealed that the composition of the microcosms derived from the saliva of donor A was dissimilar from the inoculum at all time points (Fig. 1a). A different pattern was observed for the composition of the microcosms derived from the saliva of donor B. There was a more gradual shift from the composition of the inoculum with time (Fig. 1b). For donor A, the diversity of the nitrate microcosms was significantly higher (*p* = 0.026) compared to the control microcosms (Fig. S3A), while for donor B, the diversity of the microcosms was significantly (*p* = 0.010) higher in the control group compared to the nitrate group (Fig. S3B). One-way PERMANOVA indicated that there was no significant difference in microbiome composition between the treatments per week for donor A (Table S2), in contrast to donor B, where the microbial composition of the microcosm was significantly different between the treatments at each week.

### Genera

**Fig. 2:**
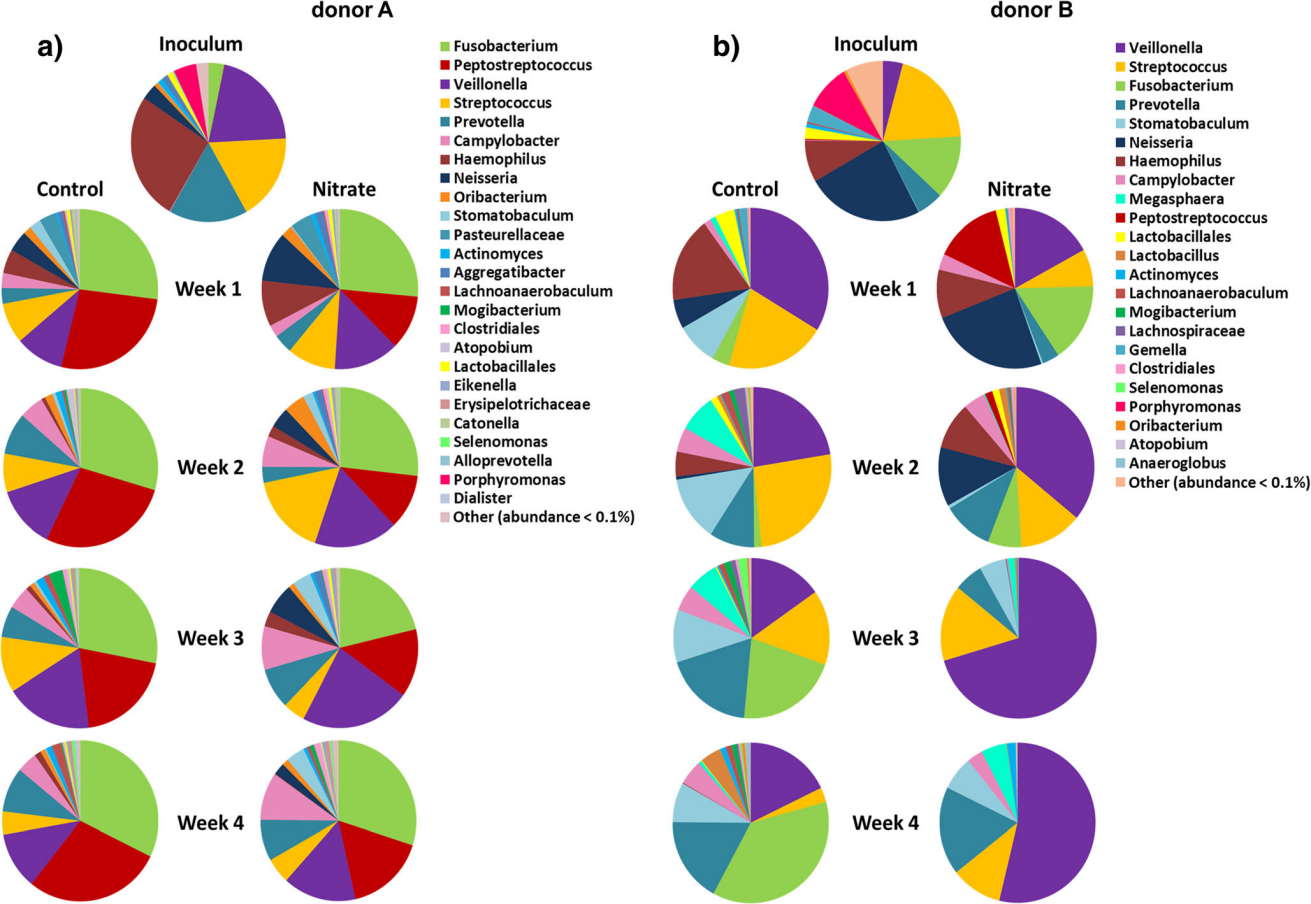
The relative abundance of genera for donor A (**a**) and donor B (**b**) per time point and treatment. Pie charts based on the average abundance of the most prevalent genera in the inoculum and per week, per treatment

The five most abundant genera in the inoculum (saliva) of donor A were *Haemophilus* (26.1 %), *Veillonella* (20.8 %), *Streptococcus* (17.7 %), *Prevotella* (16.4 %), and *Porphyromonas* (4.8 %) (Fig. 2a). The five most abundant genera in the inoculum of donor B were *Neisseria* (24.3 %), *Streptococcus* (19.9 %), *Fusobacterium* (12.9 %), *Porphyromonas* (9.2 %), and *Haemophilus* (8.4 %) (Fig. 2b).

To determine which genera were differentially abundant between the treatments, LEfSe [51] was used.

The genera that were most abundant in the inoculum of donor A were not differentially abundant between the two treatments at any of the time points in the microcosms (Fig. S4). For donor B, *Neisseria* was significantly more abundant in the nitrate group compared to the control group in weeks 1, 2, and 3. *Fusobacterium* was significantly more abundant in the nitrate group compared to the control group in the first week and was significantly more abundant in the control group in the last 2 weeks. *Haemophilus* was significantly more abundant in the control group in the last week.

### OTUs

**Fig. 3:**
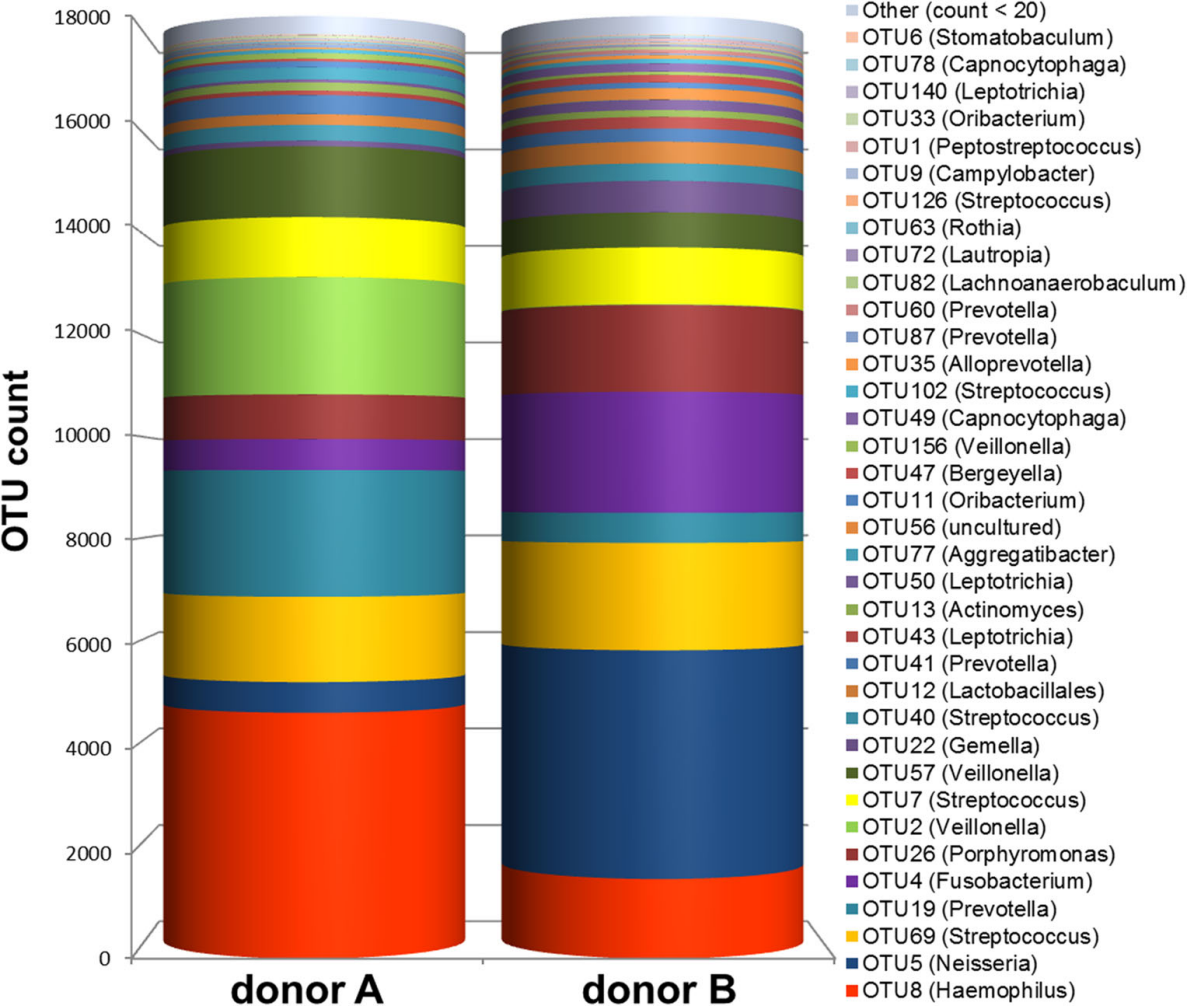
OTUs in the inoculum of each donor. The bar chart shows the total count of the most abundant OTUs in the inoculum of donor A and donor B. Other (count < 20) is the sum of all OTUs that were counted less than 20 times

The OTUs that dominated the inoculum of donor A were OTU8 (*Haemophilus*, 26.1 %), OTU19 (*Prevotella*, 13.5 %), OTU2 (*Veillonella*, 12.5 %), OTU69 (*Streptococcus*, 9.1 %), and OTU57 (*Veillonella*, 7.5 %) (Fig. 3). The inoculum of donor B was dominated by OTU5 (*Neisseria*, 24.3 %), OTU4 (*Fusobacterium*, 12.8 %), OTU69 (*Streptococcus*, 11.4 %), OTU26 (*Porphyromonas*, 9.2 %), and OTU8 (*Haemophilus*, 8.4 %) (Fig. 3).

**Fig. 4:**
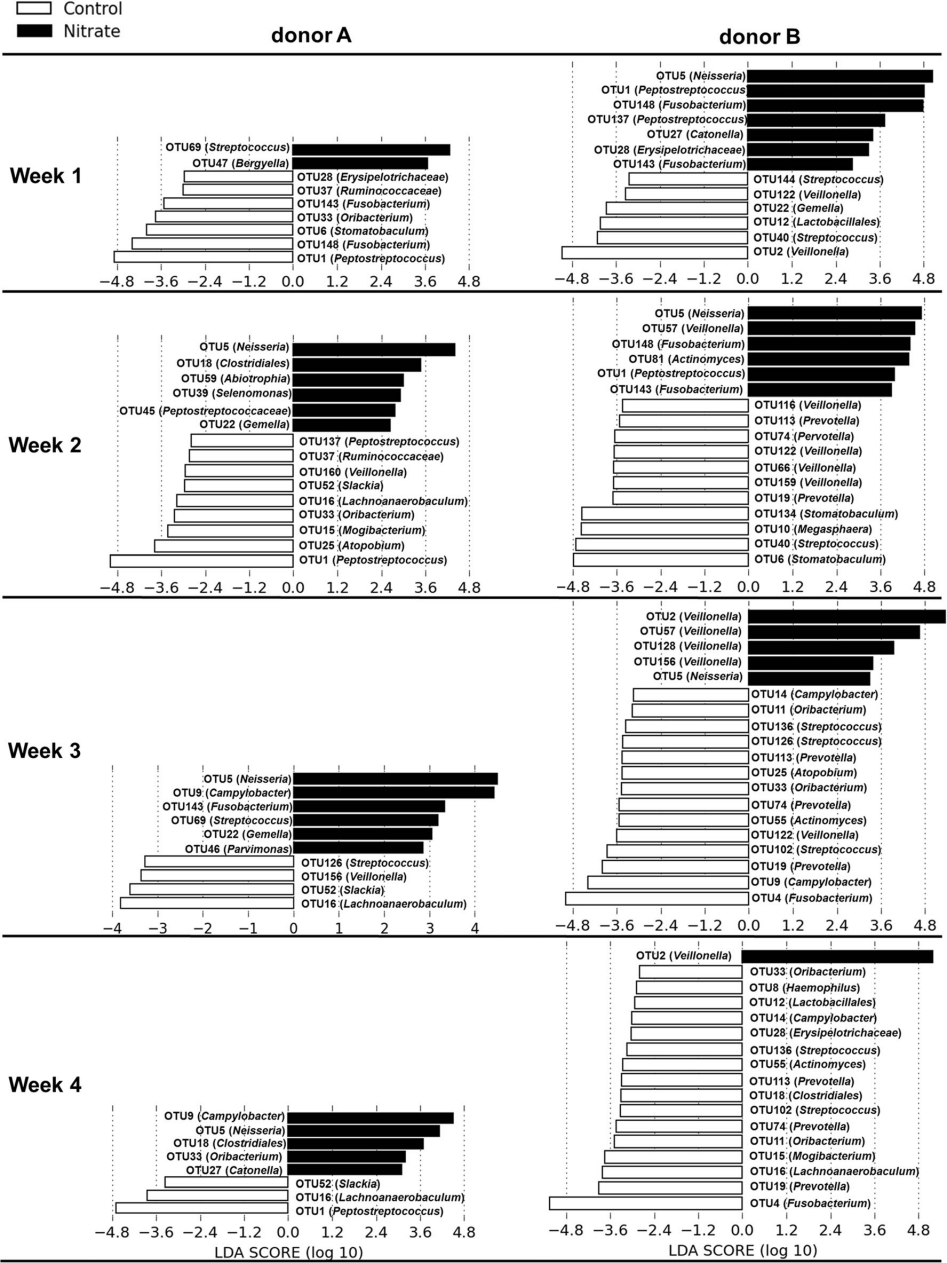
Differentially abundant OTUs between the two treatments at each time point per donor. The OTUs that were identified as differentially abundant through linear discriminant analysis effect (LEfSe) size score between the two treatments are displayed in the histogram. The *white bars* represent OTUs that were associated with the control group; the *black bars* represent OTUs that were associated with the nitrate group

LEfSe was used to determine which OTUs were differentially abundant between the two treatments at each of the four sampling weeks (Fig. 4). For donor A, OTU5 (*Neisseria*) was associated with the nitrate microcosms at weeks 2, 3, and 4. The same accounted for OTU9 (*Campylobacter*) at weeks 3 and 4. In the microcosms derived from donor B, OTU9 (*Campylobacter*) was associated with the control microcosms, although only at week 3. OTU5 (*Neisseria*) was associated with the nitrate treatment in the microcosms derived from donor B, similar to the microcosms derived from donor A, only at weeks 1, 2, and 3. At week 3, four other OTUs were associated with the nitrate treatment as well, namely OTU2, OTU57, OTU218, and OTU156 (all *Veillonella*). At week 4, only OTU2 (*Veillonella*) was associated with the nitrate treatment of the donor B-derived microcosms. In contrast, for this same donor, OTU2 (*Veillonella*) was associated with the control treatment at week 1.

OTU1 (*Peptostreptococcus*) was associated with the control microcosms derived from donor A in all but week 3, while for donor B, OTU1 (*Peptostreptococcus*) was associated with the nitrate treatment at weeks 1 and 2.

### Nitrate Reduction

**Fig. 5:**
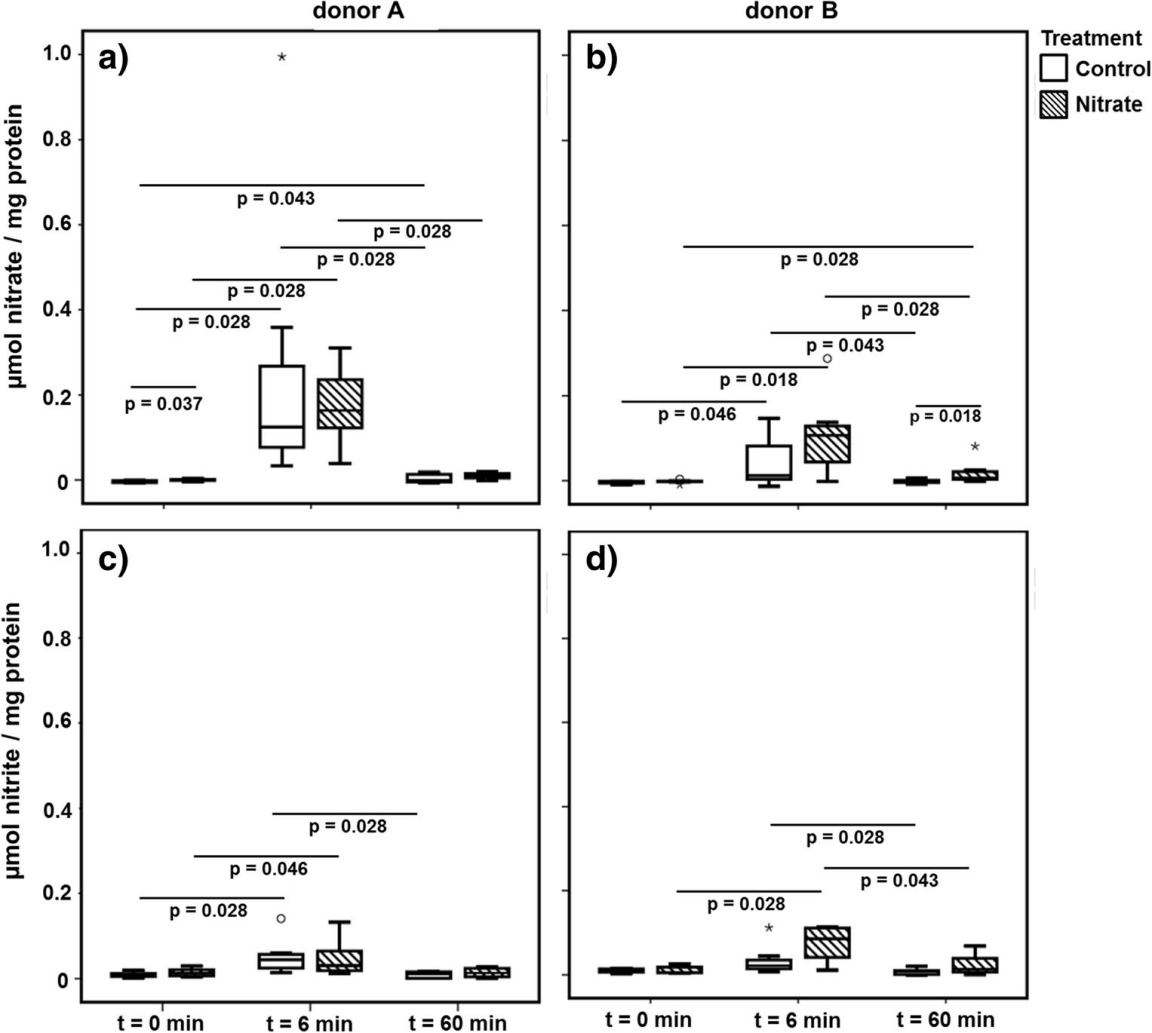
Reduction of nitrate and formation of nitrite after the addition of nitrate to the microcosms. The boxplots represent the amount of nitrate (**a**, **b**) and nitrite (**c**, **d**) in the microcosms before the addition of nitrate (*t* = 0 min) and after the addition of nitrate (*t* = 6 min and *t* = 60 min) for both treatments. The significance (*p* < 0.05) of the difference in nitrate or nitrite concentration between the time points of the same treatment was tested using the Wilcoxon signed ranks test. The significance (*p* < 0.05) of the difference in concentration between the treatments at a single time point was tested using the Mann-Whitney test. The *boxes* represent the median and interquartile range (IQR) and outliers more than 1.5× IQR are depicted by *circles* and more than 3× IQR by *stars*

A 6-min nitrate pulse was added to the MAM. As expected, the level of nitrate increased, significantly, directly after the pulse compared to the baseline (Fig. 5a, b). The level of nitrate at this time point was higher in the microcosms derived from donor A, compared to donor B. After 1 h, the nitrate concentration had decreased significantly, resembling the baseline values.

Nitrite concentrations were measured simultaneously with nitrate. In the microcosms derived from donor A, the concentration of nitrite had increased significantly directly after the 6-min nitrate pulse for both treatments (Fig. 5c), while in the microcosms derived from donor B, only in the nitrate-treated microcosms nitrite increased significantly compared to the baseline (Fig. 5d). Yet, after 1 h, the nitrite concentration in the control microcosms of both donors and nitrate-treated microcosms from donor B had significantly decreased.

In addition to measuring nitrate reduction in the microcosms, nitrate reduction assays were performed (Fig. S5). The levels of nitrate, nitrite, and ammonium were measured after 1 h of incubation. No significant differences between either aerobic or anaerobic incubation or between the treatments were found.

### Acid Formation

**Fig. 6:**
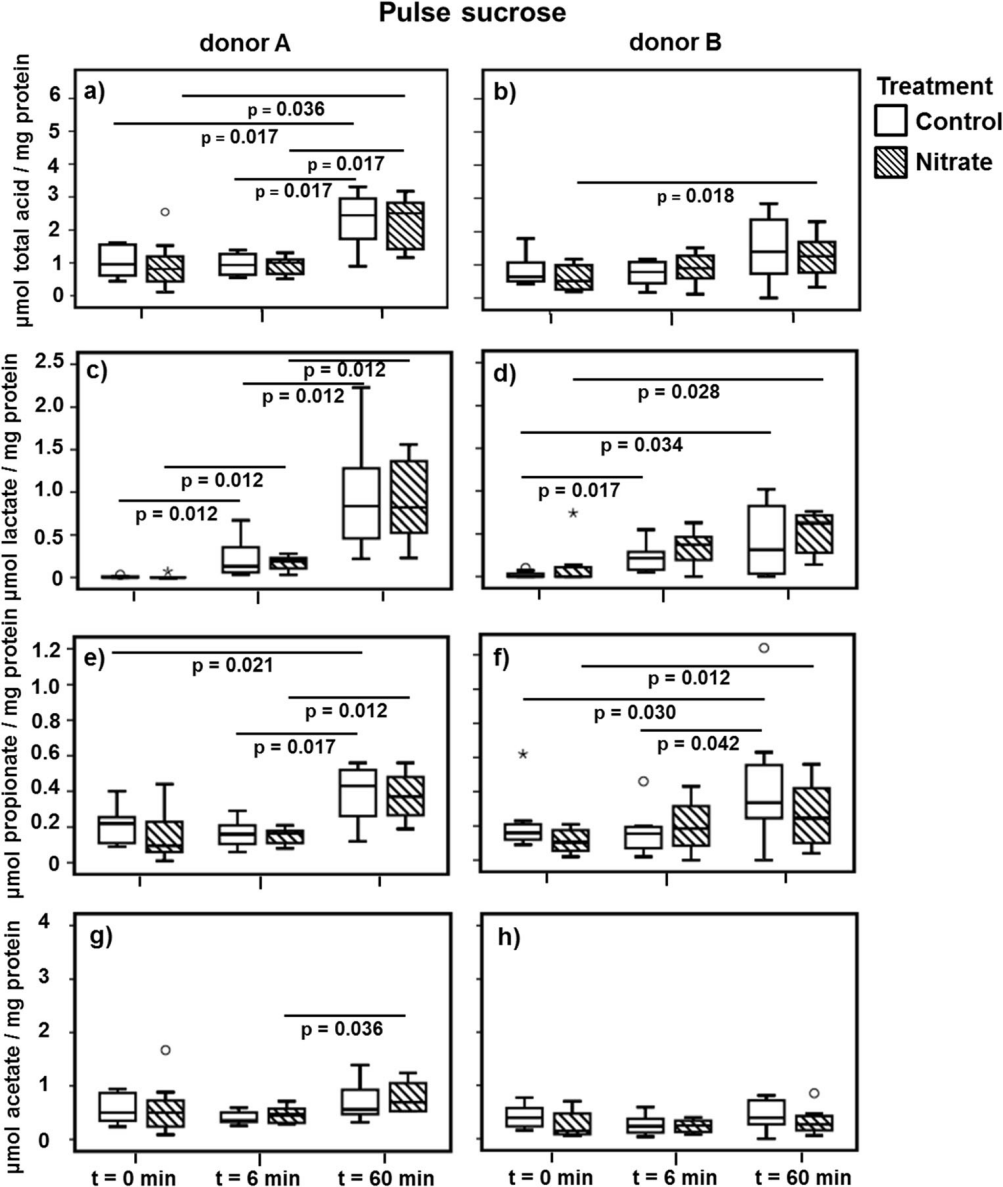
Short chain fatty acid concentrations before and after the addition of sucrose to the microcosms. The boxplots represent the concentration of all short chain fatty acids combined (**a**, **b**) (including succinate, formate, and butyrate), lactate (**c**, **d**), propionate (**e**, **f**), and acetate (**g**, **h**) before (*t* = 0 min) and after (*t* = 6 min and *t* = 60 min) the addition of sucrose. The significance (*p* < 0.05) of the difference in acid concentration between the time points of the same treatment was tested using the Wilcoxon signed ranks test. The *boxes* represent the median and interquartile range (IQR), and outliers more than 1.5× IQR are depicted by *circles* and more than 3× IQR by *stars*

The concentration of short chain fatty acids was measured before (*t* = 0 min) and after (*t* = 6 min and *t* = 60 min) the addition of sucrose or nitrate pulse to the microcosms. When sucrose was added to the microcosm, the total short chain fatty acid concentration had increased significantly 1 h after the sucrose pulse in the microcosms derived from donor A, irrespective of the treatment group (Fig. 6a), while in the microcosms derived from donor B, this had only occurred in the nitrate group (Fig. 6b). The concentration of lactate increased 6 min after the addition of sucrose and was highest at *t* = 60 min compared to *t* = 0 min and *t* = 6 min for both donors (Fig. 6c, d). Similar to lactate, the concentration of propionate was the highest at *t* = 60 min after sucrose addition (Fig. 6e, f). The addition of sucrose hardly affected the acetate concentration (Fig. 6g, h) and did not affect the butyrate concentration.

**Fig. 7:**
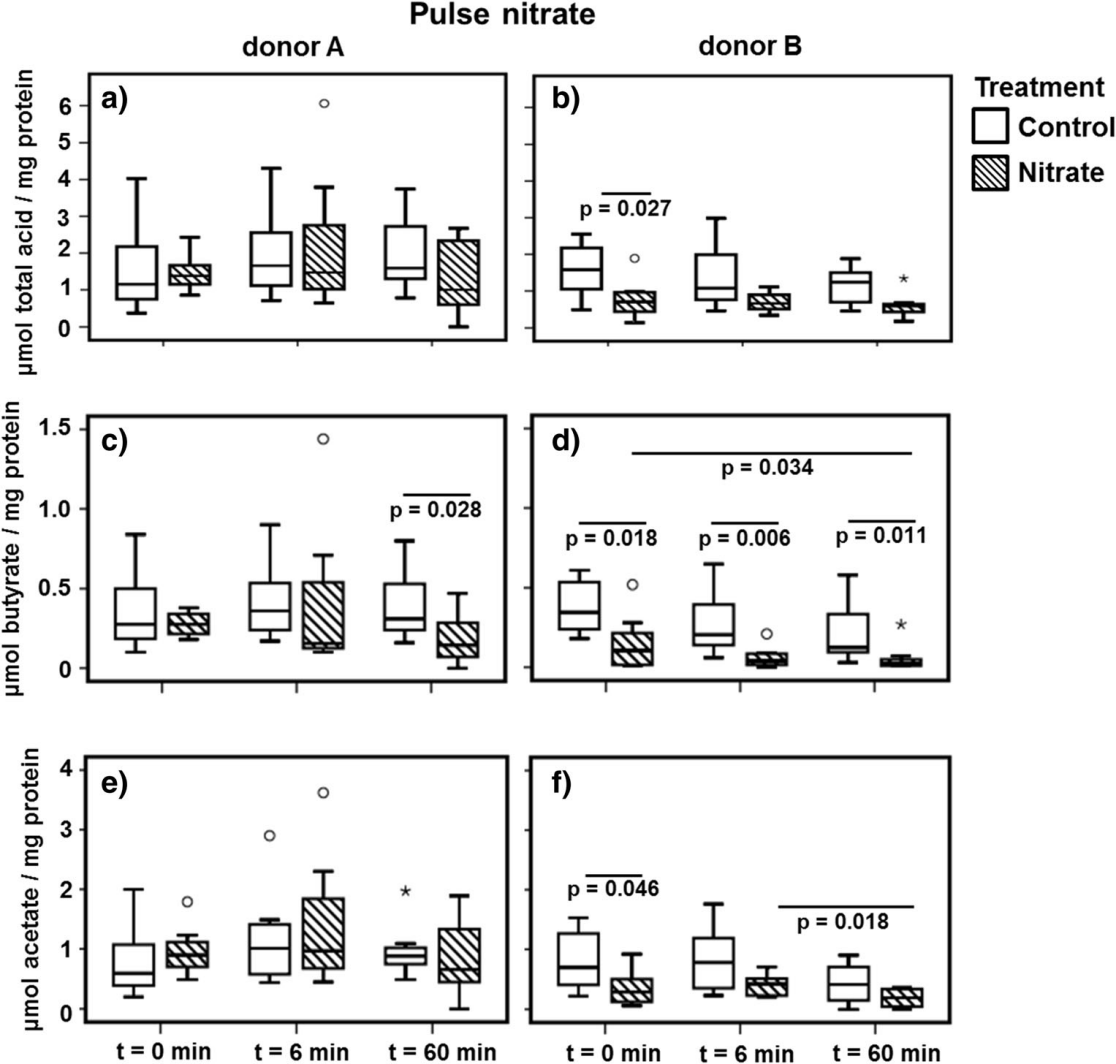
Short chain fatty acid concentrations before and after the addition of nitrate to the microcosms. The boxplots represent the concentration of all short chain fatty acids combined (**a**, **b**) (including lactate, succinate, formate, and propionate), butyrate (**c**, **d**), and acetate (**e**, **f**) before (*t* = 0 min) and after (*t* = 6 min and *t* = 60 min) the addition of nitrate. The significance (*p* < 0.05) of the difference in acid concentration between the time points of the same treatment was tested using the Wilcoxon signed ranks test. The significance (*p* < 0.05) of the difference in acid concentration between the treatments at a single time point was tested using the Mann-Whitney test. The *boxes* represent the median and interquartile range (IQR), and outliers more than 1.5× IQR are depicted by *circles* and more than 3× IQR by *stars*

The total short chain fatty acid concentration after the nitrate pulse showed a different trend for each donor (Fig. 7a, b). While in donor A microcosms, there was no difference between the control and nitrate groups, in donor B microcosms, samples from the nitrate group had a lower total short chain fatty acid concentration compared to the control group.

The concentration of butyrate after the addition of nitrate, for donor A, was significantly lower in the nitrate microcosms compared to the control microcosms at *t* = 60 min (Fig. 7c). For donor B, the concentration of butyrate was significantly lower in the nitrate group compared to the control group at all time points (Fig. 7d). The concentration of acetate after the addition of nitrate was consistently lower in the Nitrate group microcosms, compared to the Control microcosms of donor B (Fig. 7f). Compared to donor B, the concentration of acetate in the Nitrate group of donor A, was relatively high (Fig. 7e). The addition of nitrate had no significant effect on the concentration of propionate and lactate.

Furthermore, a sucrose metabolism assay was performed (Fig. S6). The total concentration of acid increased significantly 10 min after the addition of sucrose for both donors and treatments. In agreement with the total acid concentration, most individual short chain fatty acids increased in concentration after the addition of sucrose to the system.

During the sucrose metabolism assay, the pH was measured. The pH of the nitrate group was significantly higher than the pH of the control group at the start of the assay for donor A (Fig. S7). After the 10-min incubation with sucrose, the pH had dropped significantly (~2 units) for both treatments and donors.

The concentration of phosphate was measured simultaneously with the fatty acids in the sucrose metabolism assay. The concentration of phosphate had increased significantly after 10 min in the control group of both donors compared to the start of the assay (Fig. S8).

## Discussion

We found that the addition of nitrate to microcosms originating from human saliva has an influence on microbial composition as well as acid production. The largest difference, however, seemed to be influenced by the origin of the microcosms, i.e., the donors.

Two different inocula resulted in different types of microcosms. Especially the composition of the microcosms but also the nitrate reduction potential developed differently depending on the donors. Differences in nitrate reduction activity in the oral cavity among individuals have been observed before [4, 13, 27, 52].

For both donors, there was a ~50 % reduction in the number of OTUs in the microcosms compared to the inoculum. One of the limitations of using a model system is that it can never fully represent the original microbiome, since the circumstances will always be different from the in vivo situation. Therefore, certain species may not survive or may decrease strongly in abundance. For example, the composition of the growth medium can play a selective role in the survival of species [53–55]. In addition, certain anaerobic species might not have survived the inoculation procedure, e.g., the genus *Porphyromonas* was abundant in the inoculum of both donors, yet it could not be detected in the biofilms. The members of this anaerobic genus [56] might not have survived the aerobic inoculation procedure. However, members of the anaerobic *Veillonella* genus did survive the inoculation procedure and were among the dominant members in the microcosms.

For donor A, the microcosm composition differed from the inoculum at week 1, yet the development of the microcosms was quite similar whether nitrate was added or not. In contrast, the microcosms from donor B still demonstrated a compositional resemblance to the inoculum at week 1, yet the compositional difference between the two treatments was significant. This would suggest a rather strong compositional resilience for the microcosms derived from donor A, indicating that the dominant taxa could adapt to both situations they were challenged with, although it could also indicate that these taxa prefer other components of the medium and “ignore” the nitrate.

However, there were OTUs in the microcosms derived from donor A that were associated with the nitrate treatment, e.g., OTU5 (*Neisseria cinerea*). *N. cinerea* ATCC 14685 is known to possess nitrite reductase, nitric oxide reductase, and nitrous oxide reductase genes, although it does not seem to possess nitrate reductase genes [57].

OTU5 was also associated with the nitrate-treated microcosms derived from donor B. Yet, OTUs that were identified as members of the genus *Veillonella* were predominant in these microcosms. Interestingly, OTUs designated as *Veillonella* were associated with the control group during the first 2 weeks, while at weeks 3 and 4 (mostly), different OTUs that were also identified as *Veillonella* were associated with the nitrate group. Members of the genus *Veillonella* are known nitrate reducers [13, 58].

Some of the OTUs were present in the microcosms of both donors, yet associated with a different treatment, e.g., OTU1 (*Peptostreptococcus*) and OTU9 (*Campylobacter*). This indicates that the presence or absence of these taxa depends more on their preferred interactions with the microbial community than on the treatment.

The effect of salivary nitrate on the acidity in the oral cavity has been investigated before by, among others, Li et al. [27]. They found that under anaerobic conditions, with glucose, the pH of saliva was higher in the presence of nitrate and nitrite compared to the absence of these two compounds. In their paper, they suggest possible mechanisms underlying their observations, namely, scavenging of acids, the repression of acid fermentation, or increased alkali production. In our study, we did not observe a difference in ammonium production after the microcosms were incubated with nitrate either aerobically or anaerobically, between the two treatments and donors. Neither did we observe a difference in nitrate reduction or nitrite production in the same assay. Why we did not observe any differences is unclear. It is possible that the incubation time of the assay was too long or the right carbon source was not present.

When nitrate was added to the system, the level of nitrate within the microcosms increased, as expected. Additionally, the level of nitrate was slightly higher in the microcosms that received nitrate constantly. Remarkable was that the level of nitrate in the microcosms of donor A appeared to be higher than that of donor B after 6 min. Possibly, the nitrate reduction activity in the microcosms of donor B commenced faster compared to that of donor A. After 6 min, nitrite formation had started in both the control and Nitrate microcosms of donor A and the nitrate group of donor B. Yet, it is unclear what happens to the nitrate in the control microcosms of donor B, e.g., whether it is used in a different pathway where no nitrite is formed.

Moreover, we performed an acidification assay and did not observe a difference in pH between the two treatments after sucrose was added. The contradiction to the findings by Li et al. [27] might be explained by the fact that we did not add nitrate to this assay. In addition, this assay was performed aerobically. However, Xie et al. [59] found that, under anaerobic circumstances, the presence of nitrate in wastewater did not inhibit acidogenesis, yet it changed the fermentation metabolites.

Indeed, we observed the presence of diverse short chain fatty acids and found some differences in concentration between donors and treatments, especially after the addition of nitrate.

The concentration of butyrate decreased in time in both groups of donor B. Yet, the decrease was much more pronounced in the nitrate group. The association between nitrate and butyrate has been made before; the expression of a periplasmic nitrate reductase was higher when bacteria were grown on a reduced carbon source, such as butyrate [60, 61]. Xie et al. [59] observed that butyrate was the preferred carbon source during nitrate reduction in wastewater, which in an anaerobic acidification reactor led to an almost complete depletion of butyrate. In contrast to the study by Xie et al. [59], we did not observe an increase in acetate in the presence of nitrate.

The production of lactate after sucrose addition was similar in both treatments and donors. The microbial formation of lactate in the oral cavity is one of the processes often associated with caries [62]. Likely, most lactate producers are not capable of nitrate reduction; hence, the presence of nitrate does not influence their metabolism (suggested by Li et al. [27]). Moreover, metabolic differences between lactate producers and nitrate reducers might rule out competition between the respective species. Nonetheless, nitrate reduction can take place when lactate is the sole carbon source, although it can take over 18 h before this reaction is observed [63].

The reduction of nitrate to nitrite in the oral cavity is suggested to be a defense mechanism against caries [26]. Under acidic conditions, nitrite can be converted to nitric oxide, which is, among others, an antibacterial agent [7, 64]. This conversion would take place in the vicinity of lactate-producing, thus cariogenic, bacteria and slow their growth or even kill them [26, 28]. Although we did not measure nitric oxide concentrations in this experiment, we did measure reduction of nitrate to nitrite, which in turn can be converted to nitric oxide. Yet, we did not observe a decrease in lactate formation. This could indicate that no nitric oxide was formed in this experiment, or the lactate producers in these microcosms were not sensitive to nitric oxide. On the other hand, it might also indicate that the anti-caries effect of nitrate reduction works through a different, so far unknown, principle.

Interesting was the observation that in the sucrose metabolism assays, the concentration of phosphate had increased more in the control groups of both donors compared to the nitrate group. It has been demonstrated before that the concentration of phosphate increased in in vitro plaque samples after they were treated with sucrose [65]. Our experiment could not clarify the source of the phosphate, e.g., if it was released from the extracellular matrix by solubilization or if phosphate was released from the bacterial cells during fermentation. Nevertheless, it appears that nitrate somehow suppresses phosphate release, since the pH decrease was similar in this assay for both donors and treatments.

One of the drawbacks of this study is the relatively small amount of samples for the biochemical analyses. Considering pulse (sucrose or nitrate), treatment (control or nitrate), and donor (A or B), there were only two samples for each possible configuration per week (in contrast to the DNA samples that were taken twice a week only at *t* = 0 min and the pulse could be left out). Therefore, we could not determine the biochemical changes in time, as was done for the DNA-based samples.

Moreover, we did not measure the ability to reduce nitrate or metabolize sucrose in the inocula. These data might have provided more insight in the development of the microcosms. As clearly shown, the microbial communities derived from different donors develop differently. One appears to be compositionally resilient against nitrate supplementation, while the other adapts both compositionally and metabolically. A similar experiment should be performed, using inocula from more donors to see if these types of microcosm development are common and if they can be translated to an in vivo situation, in regard to the growing interest in the potential health benefits of nitric oxide formed as a result of nitrate reduction in the oral cavity. In addition, nitrate supplementation does affect the concentration of certain short chain fatty acids. An experiment where nitrate and sucrose are supplied at the same moment should be performed to know if this would influence the formation of lactate. Moreover, if nitrate reduction in the oral cavity of an individual is very low, supplementation with a specific carbon source or even molybdenum might be interesting.

In short, a lot remains to be discovered about nitrate in the oral cavity, yet it certainly influences microbial composition as well as biochemistry.

## Electronic supplementary material

Below is the link to the electronic supplementary material.

Table S1 (DOCX 14 kb)

Table S2 (DOCX 14 kb)

Fig. S1 Schematic overall configuration of the multi-plaque artificial mouth (MAM) system (**a**). Adapted from Wong L (2001) Mineralisation in dental plaque model systems. PhD Thesis, University of Otago, Dunedin, New Zealand. Photograph of the operational MAM (**b**) (GIF 265 kb)

High Resolution (TIF 3160 kb)

Fig. S2 Sampling scheme of the experiment. All microcosms (stations) received a continuous supply of defined mucin medium (DMM) supplemented with trace element solution DSMZ SL-4. The hatched circles represent the microcosms that received a continuous supply of 1 mM nitrate in addition to DMM. All microcosms received eight 6 min pulses for 10 % w/v sucrose per 24 h. The pulses started at 17:00 h and ended at 07:00 h. In addition, all microcosms received pulses of nitrate and sucrose once a week, pulses were given on separate days, to measure nitrate reduction and acid production (GIF 72 kb)

High Resolution (TIF 891 kb)

Fig. S3 Shannon diversity index. The Shannon diversity index was calculated using PAST. Statistical significance (p < 0.05) was determined using the Wilcoxon Signed Ranks Test (GIF 25 kb)

High Resolution (TIF 321 kb)

Fig. S4 Differentially abundant genera between the two treatments at each time point per donor. The genera that were identified as differentially abundant through linear discriminant analysis effect (LEfSe) size score between the two treatments are displayed in the histogram. The white bars represent genera that were associated with the Control group; the black bars represent genera that were associated with the Nitrate group (GIF 96 kb)

High Resolution (TIF 1109 kb)

Fig. S5 The concentration of nitrate, nitrite and ammonium after the nitrate reduction assay. The concentrations of nitrate (**a** and **b**), nitrite (**c** and **d**) and ammonium (**e** and **f**) were measured 1 h after adding 1 mM nitrate to the cell pellets. The samples were incubated aerobically and anaerobically (GIF 78 kb)

High Resolution (TIF 1083 kb)

Fig. S6 Short chain fatty acid concentrations at the start and end point of the sucrose metabolism assay. The boxplots represent the concentration of all short chain fatty acids combined (**a** and **b**), lactate (**c** and **d**), succinate (**e** and **f**), acetate (**g** and **h**), propionate (**i** and **j**) and butyrate (**k** and **l**) at the start (t =0 min), and end (t = 10 min) of the assay. The significance (p < 0.05) of the difference in acid concentration between the time points of the same treatment was tested using the Wilcoxon Signed Ranks Test. The boxes represent the median and interquartile range (IQR), outliers more than 1.5× IQR are depicted by ○, and more than 3× IQR by ★ (GIF 70 kb)

High Resolution (TIF 835 kb)

Fig. S7 The pH at the start and end point of the sucrose metabolism assay. The significance (p < 0.05) of the difference in pH between the time points of the same treatment was tested using the Wilcoxon Signed Ranks Test. The significance (p < 0.05) of the difference in pH between the treatments at a single time point was tested using the Mann-Whitney Test. The boxes represent the median and interquartile range (IQR), outliers more than 1.5× IQR are depicted by ○, and more than 3× IQR by ★ (GIF 24 kb)

High Resolution (TIF 273 kb)

Fig. S8 The concentration of phosphate at the start and end point of the sucrose metabolism assay. The concentration of phosphate was measured at the baseline (t = 0 min) and ten min (t = 10 min) after the addition of sucrose to the cell pellets. The significance (p < 0.05) of the difference in phosphate concentration between the time points of the same treatment was tested using the Wilcoxon Signed Ranks Test. The boxes represent the median and interquartile range (IQR), outliers more than 1.5× IQR are depicted by ○, and more than 3× IQR by ★ (GIF 25 kb)

High Resolution (TIF 315 kb)
