# Supplementary material for: Nitrate and the Origin of Saliva Influence Composition and Short Chain Fatty Acid Production of Oral Microcosms
Source: Microb Ecol. 2016 May 7;72:479–92. doi: 10.1007/s00248-016-0775-z (PMC4937104; doi:10.1007/s00248-016-0775-z)
Supplement: Supplementary file 1 — (DOCX 14 kb) [file 248_2016_775_MOESM1_ESM.docx]

**Supplementary table 1:** DSMZ trace element solution

| **Trace element solution SL-4:** |  |
| --- | --- |
| EDTA | 0.50 g |
| FeSO_4_ x 7 H_2_O | 0.20 g |
| Trace element solution SL-6 (see below) | 100.00 ml |
| Distilled water | 900.00 ml |

| **Trace element solution SL-6:** |  |
| --- | --- |
| ZnSO_4_ x 7 H_2_O | 0.10 g |
| MnCl_2_ x 4 H_2_O | 0.03 g |
| H_3_BO_3_ | 0.30 g |
| CoCl_2_ x 6 H_2_O | 0.20 g |
| CuCl_2_ x 2 H_2_O | 0.01 g |
| NiCl_2_ x 6 H_2_O | 0.02 g |
| Na_2_MoO_4_ x 2 H_2_O | 0.03 g |
| Distilled water | 1000.00 ml |
