# Supplementary material for: Nitrate and the Origin of Saliva Influence Composition and Short Chain Fatty Acid Production of Oral Microcosms
Source: Microb Ecol. 2016 May 7;72:479–92. doi: 10.1007/s00248-016-0775-z (PMC4937104; doi:10.1007/s00248-016-0775-z)
Supplement: Supplementary file 2 — (DOCX 14 kb) [file 248_2016_775_MOESM2_ESM.docx]

**Supplementary table 2:** One-way PERMANOVA between the Control and Nitrate groups

|  | donor A | | donor B | |
| --- | --- | --- | --- | --- |
|  | p | F | p | F |
| Week 1 | 0.0809 | 1.932 | 0.0267 | 6.522 |
| Week 2 | 0.0604 | 2.540 | 0.0317 | 3.449 |
| Week 3 | 0.1430 | 2.134 | 0.0299 | 12.96 |
| Week 4 | 0.1712 | 1.914 | 0.0301 | 6.599 |
| Statistical significance threshold: p < 0.05 | | | | |
