# Supplementary figures and images for: Nitrate and the Origin of Saliva Influence Composition and Short Chain Fatty Acid Production of Oral Microcosms
Source: Microb Ecol. 2016 May 7;72:479–92. doi: 10.1007/s00248-016-0775-z (PMC4937104; doi:10.1007/s00248-016-0775-z)

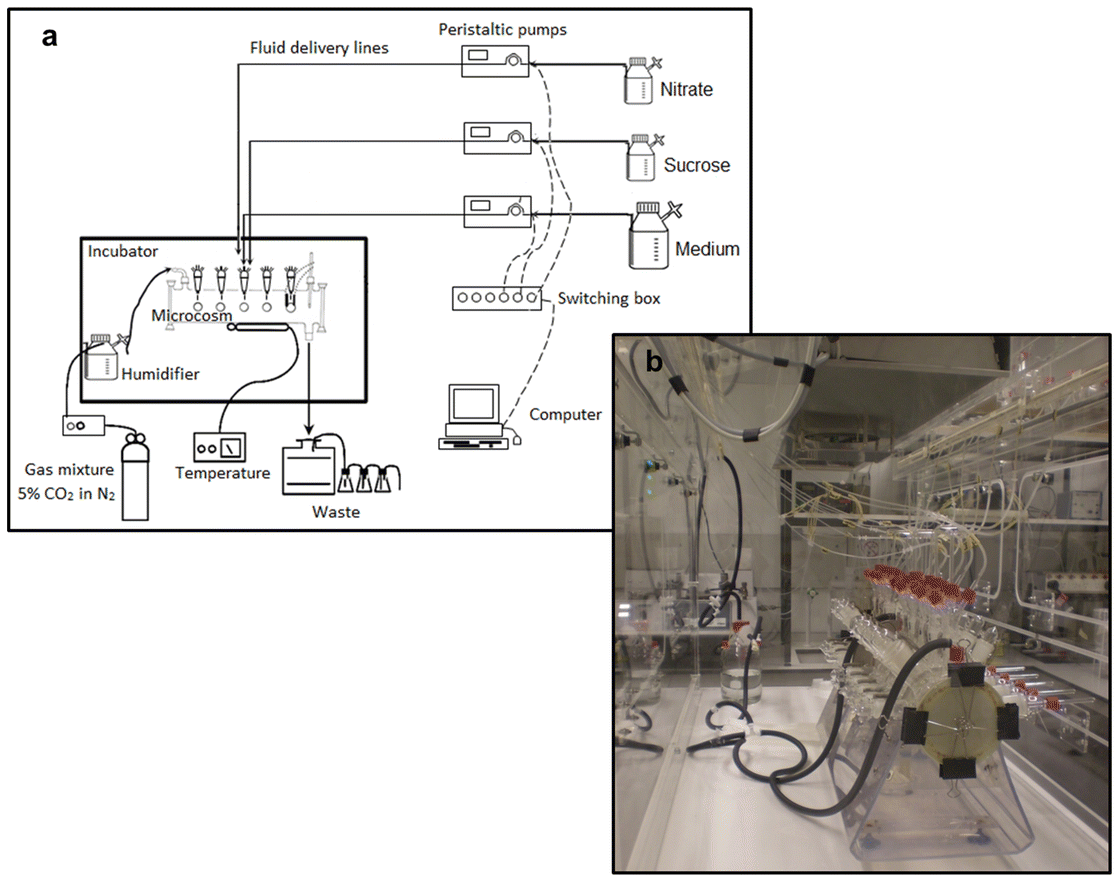

Supplement: Supplementary file 3 — Schematic overall configuration of the multi-plaque artificial mouth (MAM) system (a). Adapted from Wong L (2001) Mineralisation in dental plaque model systems. PhD Thesis, University of Otago, Dunedin, New Zealand. Photograph of the operational MAM (b) (GIF 265 kb) [file 248_2016_775_Fig8_ESM.gif]

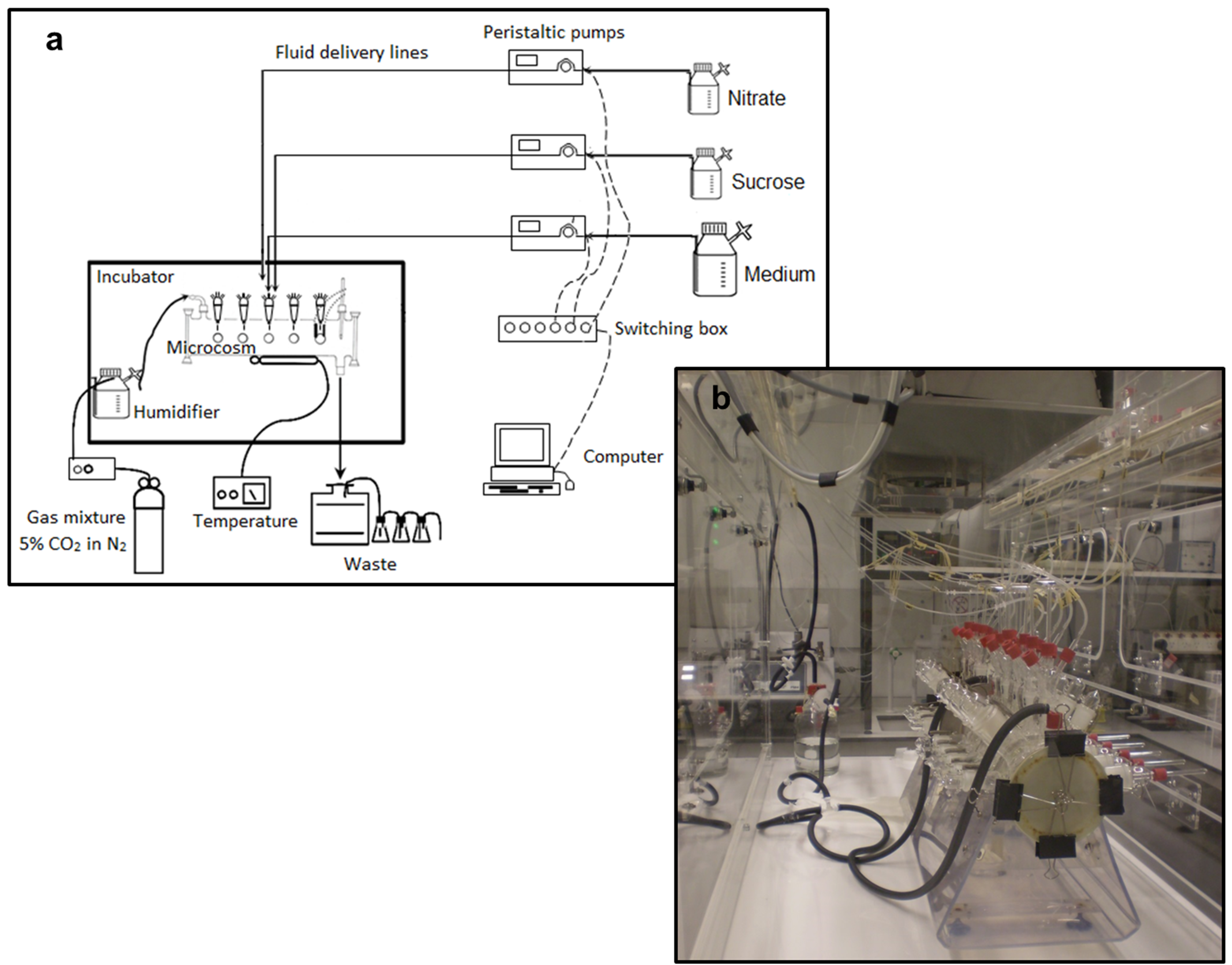

Supplement: Supplementary file 4 — High Resolution (TIF 3160 kb) [file 248_2016_775_MOESM3_ESM.tif]

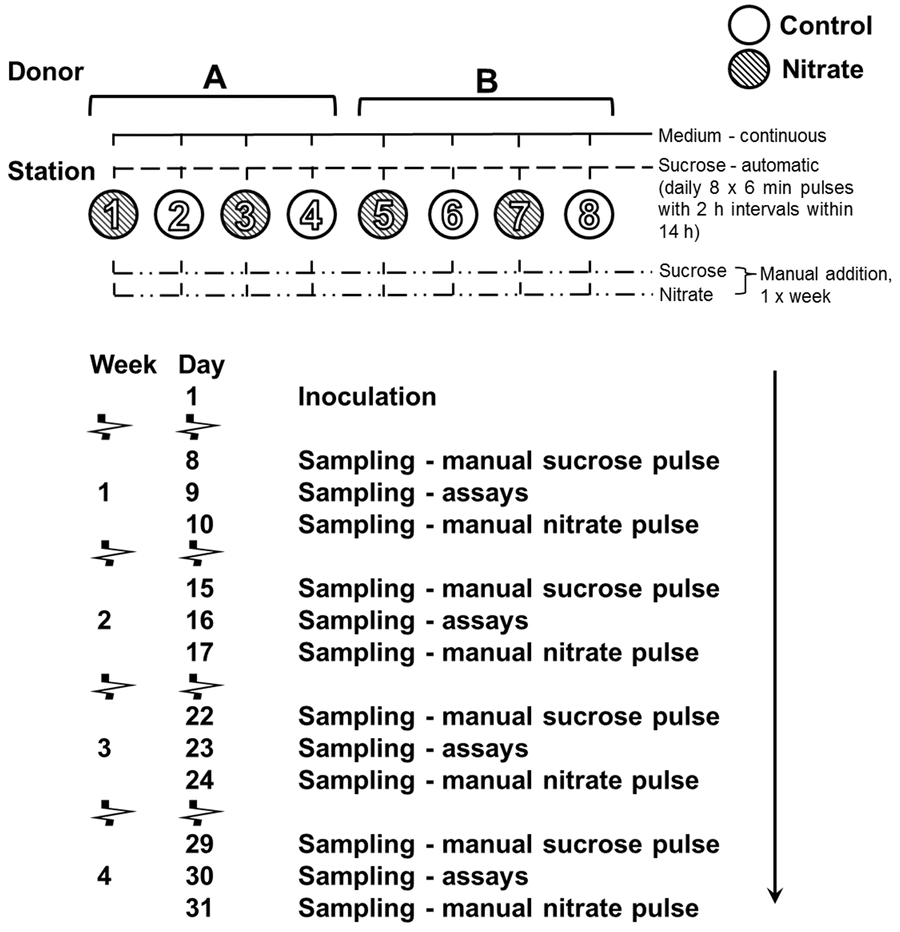

Supplement: Supplementary file 5 — Sampling scheme of the experiment. All microcosms (stations) received a continuous supply of defined mucin medium (DMM) supplemented with trace element solution DSMZ SL-4. The hatched circles represent the microcosms that received a continuous supply of 1 mM nitrate in addition to DMM. All microcosms received eight 6 min pulses for 10 % w/v sucrose per 24 h. The pulses started at 17:00 h and ended at 07:00 h. In addition, all microcosms received pulses of nitrate and sucrose once a week, pulses were given on separate days, to measure nitrate reduction and acid production (GIF 72 kb) [file 248_2016_775_Fig9_ESM.gif]

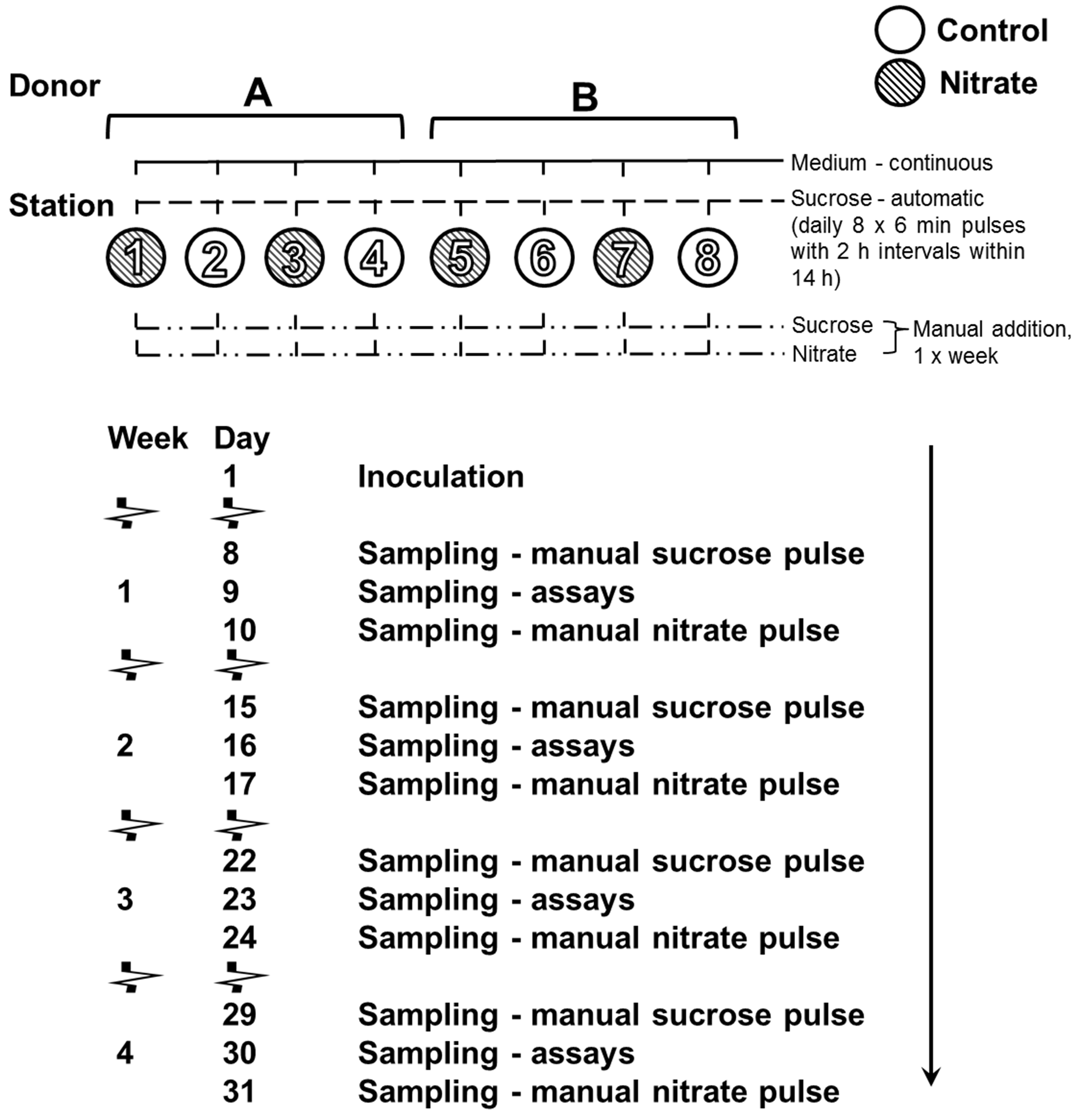

Supplement: Supplementary file 6 — High Resolution (TIF 891 kb) [file 248_2016_775_MOESM4_ESM.tif]

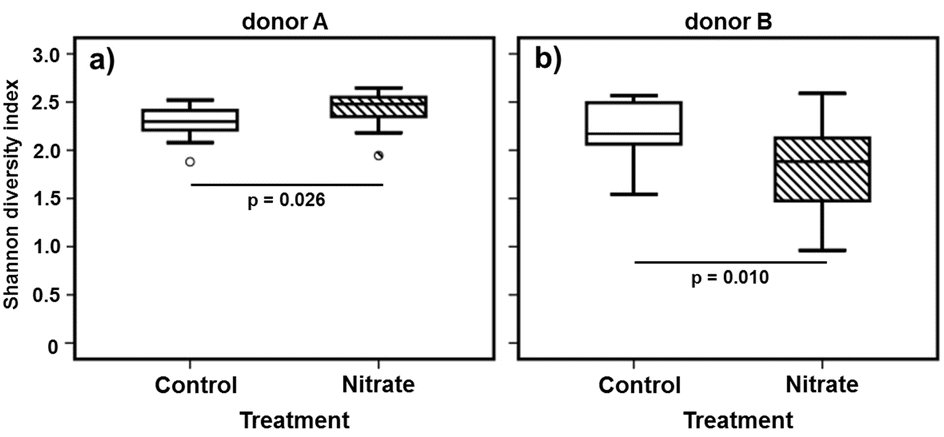

Supplement: Supplementary file 7 — Shannon diversity index. The Shannon diversity index was calculated using PAST. Statistical significance (p < 0.05) was determined using the Wilcoxon Signed Ranks Test (GIF 25 kb) [file 248_2016_775_Fig10_ESM.gif]

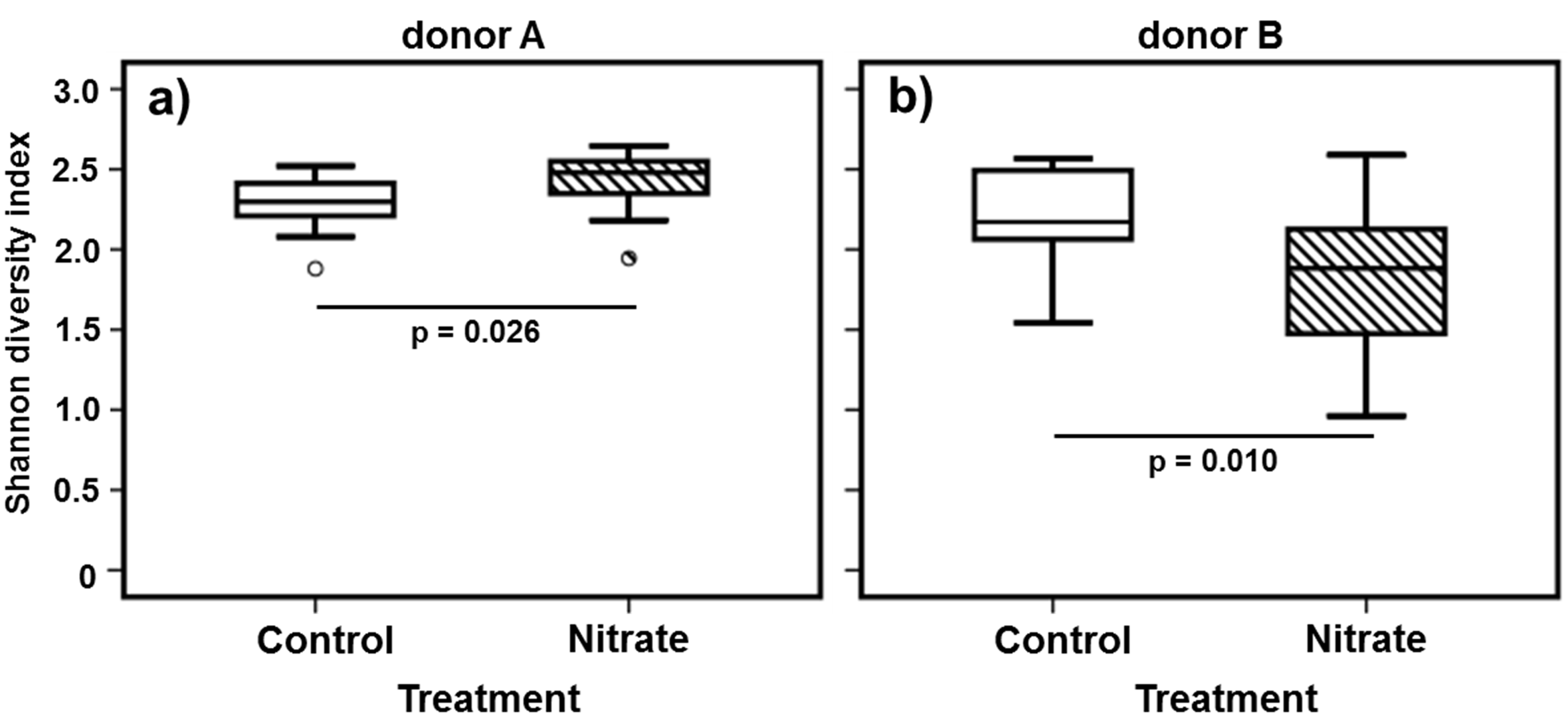

Supplement: Supplementary file 8 — High Resolution (TIF 321 kb) [file 248_2016_775_MOESM5_ESM.tif]

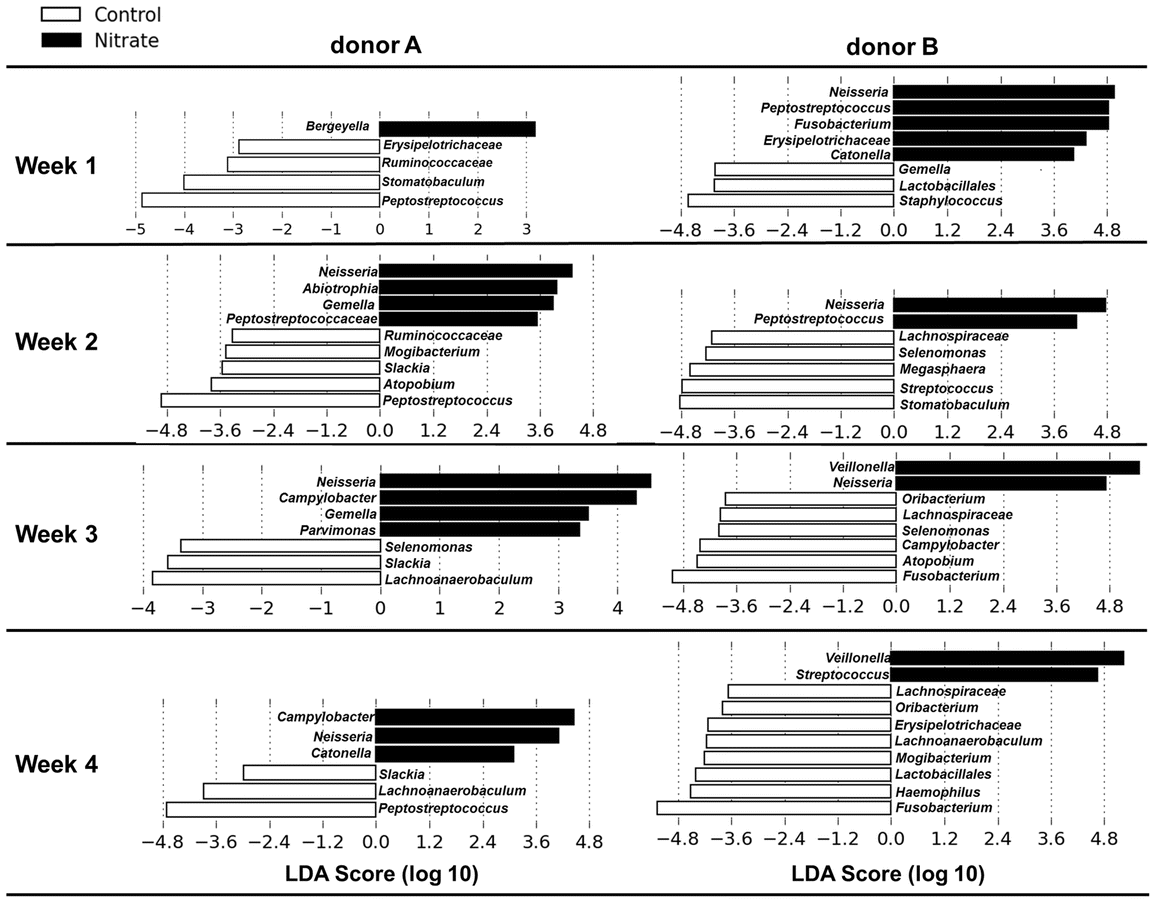

Supplement: Supplementary file 9 — Differentially abundant genera between the two treatments at each time point per donor. The genera that were identified as differentially abundant through linear discriminant analysis effect (LEfSe) size score between the two treatments are displayed in the histogram. The white bars represent genera that were associated with the Control group; the black bars represent genera that were associated with the Nitrate group (GIF 96 kb) [file 248_2016_775_Fig11_ESM.gif]

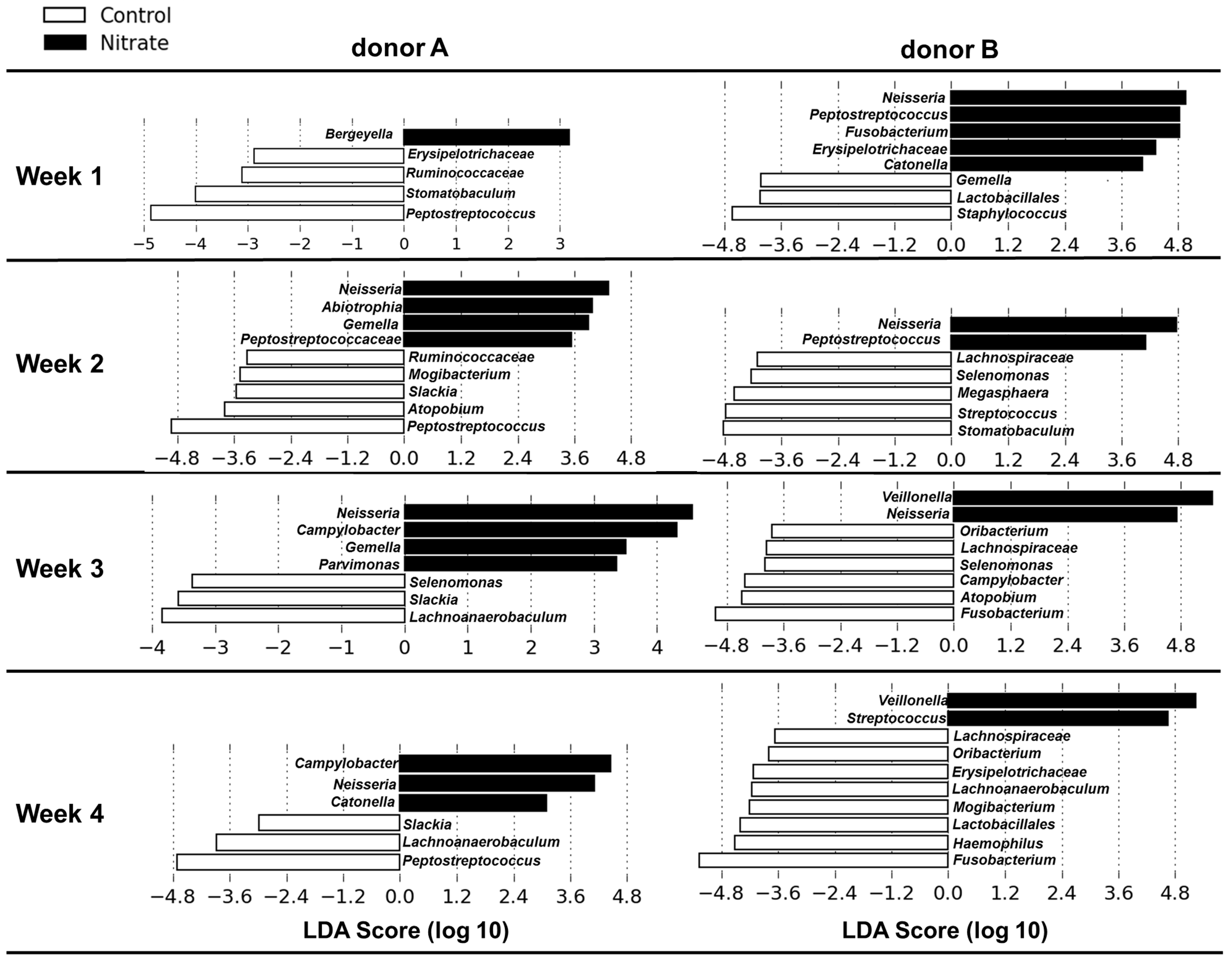

Supplement: Supplementary file 10 — High Resolution (TIF 1109 kb) [file 248_2016_775_MOESM6_ESM.tif]

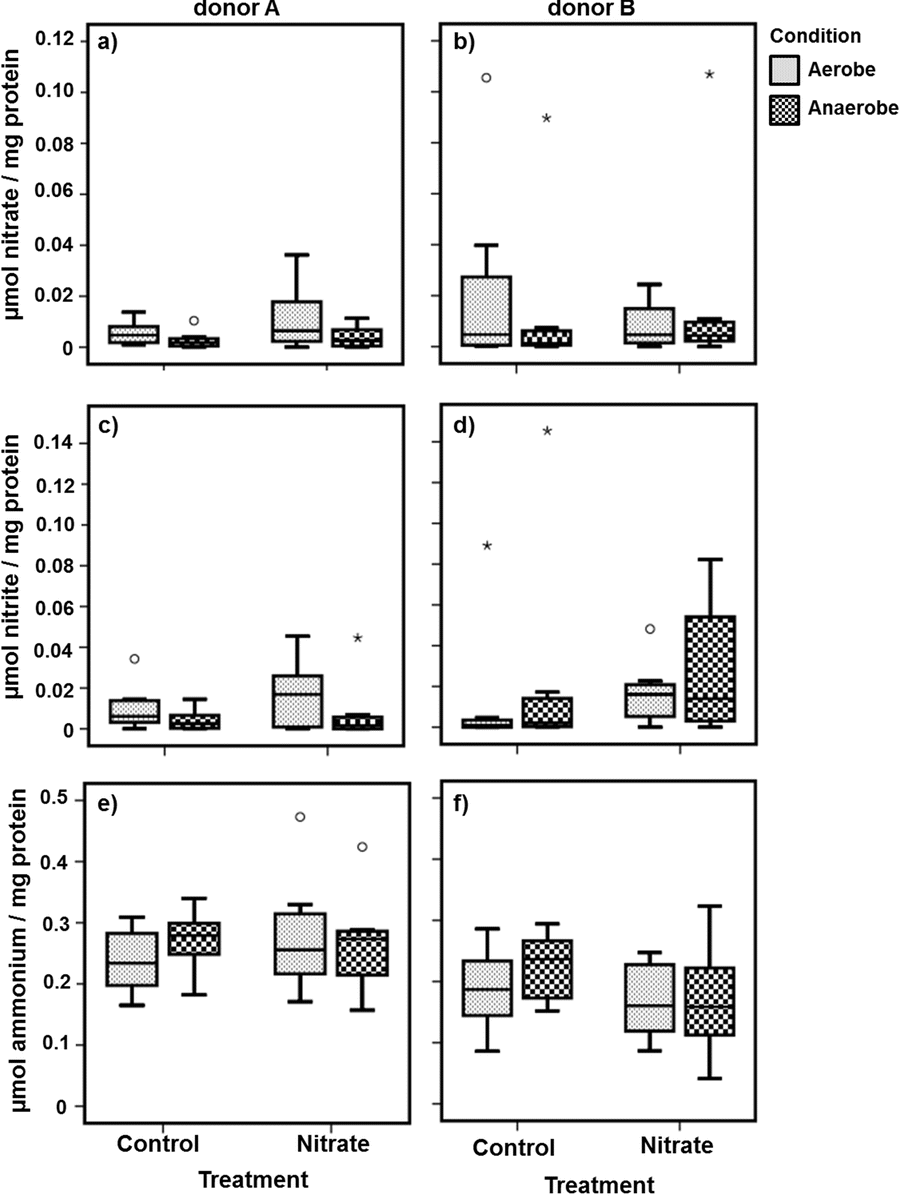

Supplement: Supplementary file 11 — The concentration of nitrate, nitrite and ammonium after the nitrate reduction assay. The concentrations of nitrate (a and b), nitrite (c and d) and ammonium (e and f) were measured 1 h after adding 1 mM nitrate to the cell pellets. The samples were incubated aerobically and anaerobically (GIF 78 kb) [file 248_2016_775_Fig12_ESM.gif]

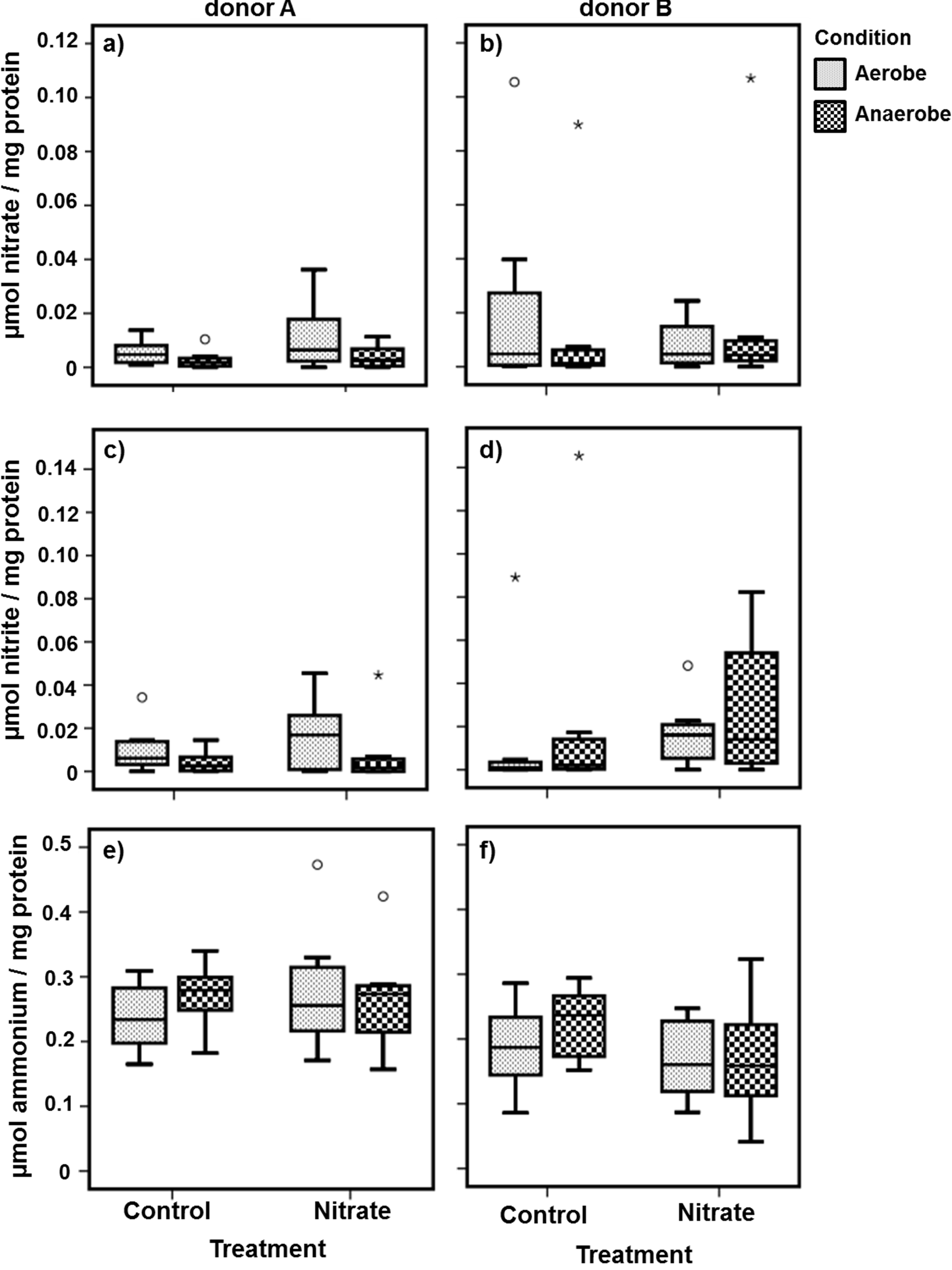

Supplement: Supplementary file 12 — High Resolution (TIF 1083 kb) [file 248_2016_775_MOESM7_ESM.tif]

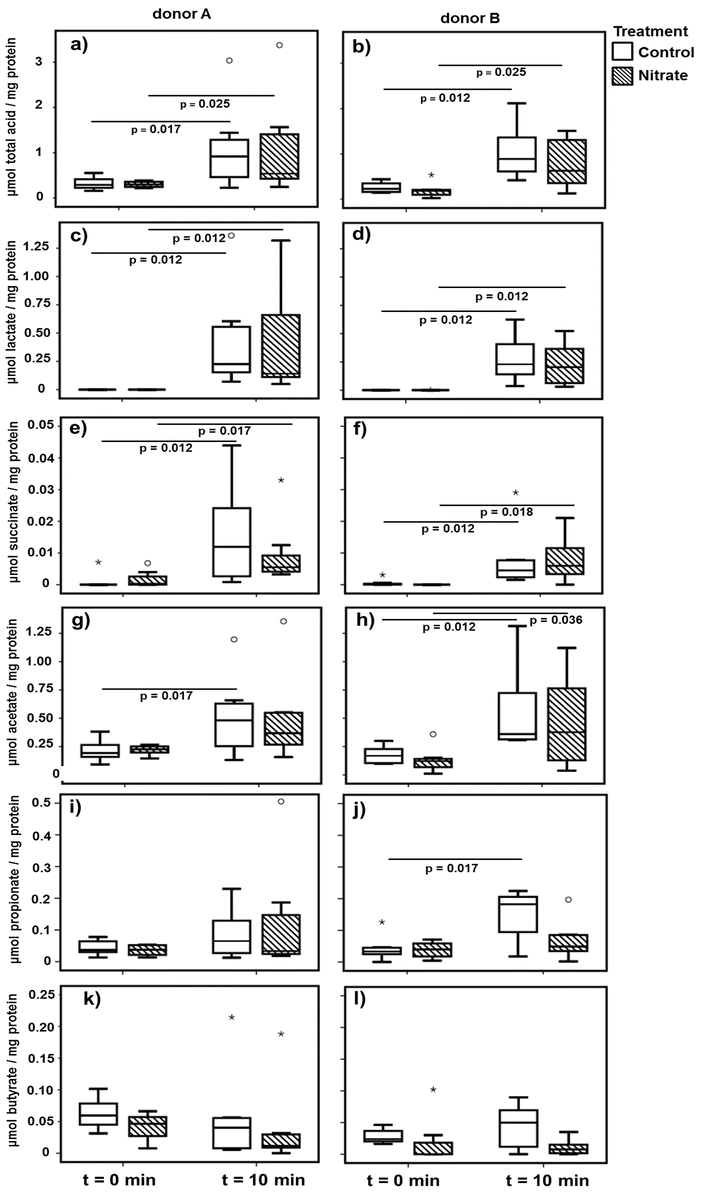

Supplement: Supplementary file 13 — Short chain fatty acid concentrations at the start and end point of the sucrose metabolism assay. The boxplots represent the concentration of all short chain fatty acids combined (a and b), lactate (c and d), succinate (e and f), acetate (g and h), propionate (i and j) and butyrate (k and l) at the start (t =0 min), and end (t = 10 min) of the assay. The significance (p < 0.05) of the difference in acid concentration between the time points of the same treatment was tested using the Wilcoxon Signed Ranks Test. The boxes represent the median and interquartile range (IQR), outliers more than 1.5× IQR are depicted by ○, and more than 3× IQR by ★ (GIF 70 kb) [file 248_2016_775_Fig13_ESM.gif]

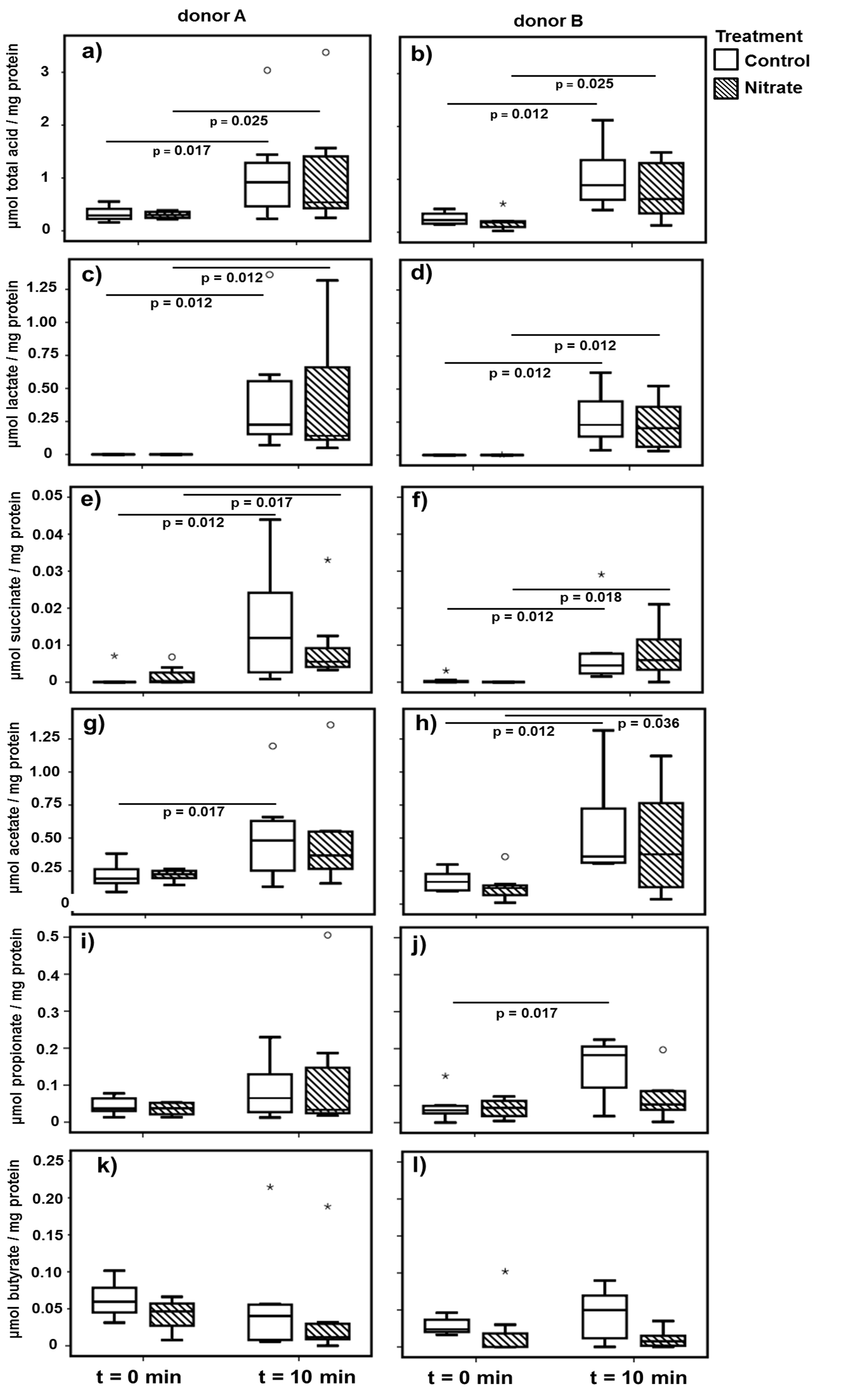

Supplement: Supplementary file 14 — High Resolution (TIF 835 kb) [file 248_2016_775_MOESM8_ESM.tif]

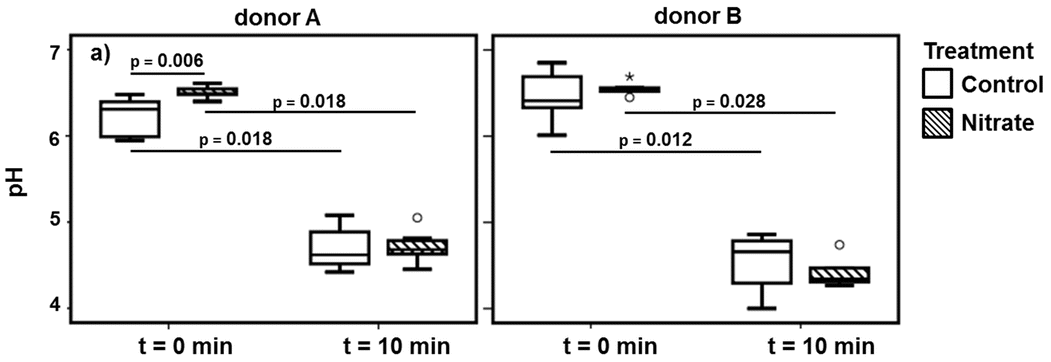

Supplement: Supplementary file 15 — The pH at the start and end point of the sucrose metabolism assay. The significance (p < 0.05) of the difference in pH between the time points of the same treatment was tested using the Wilcoxon Signed Ranks Test. The significance (p < 0.05) of the difference in pH between the treatments at a single time point was tested using the Mann-Whitney Test. The boxes represent the median and interquartile range (IQR), outliers more than 1.5× IQR are depicted by ○, and more than 3× IQR by ★ (GIF 24 kb) [file 248_2016_775_Fig14_ESM.gif]

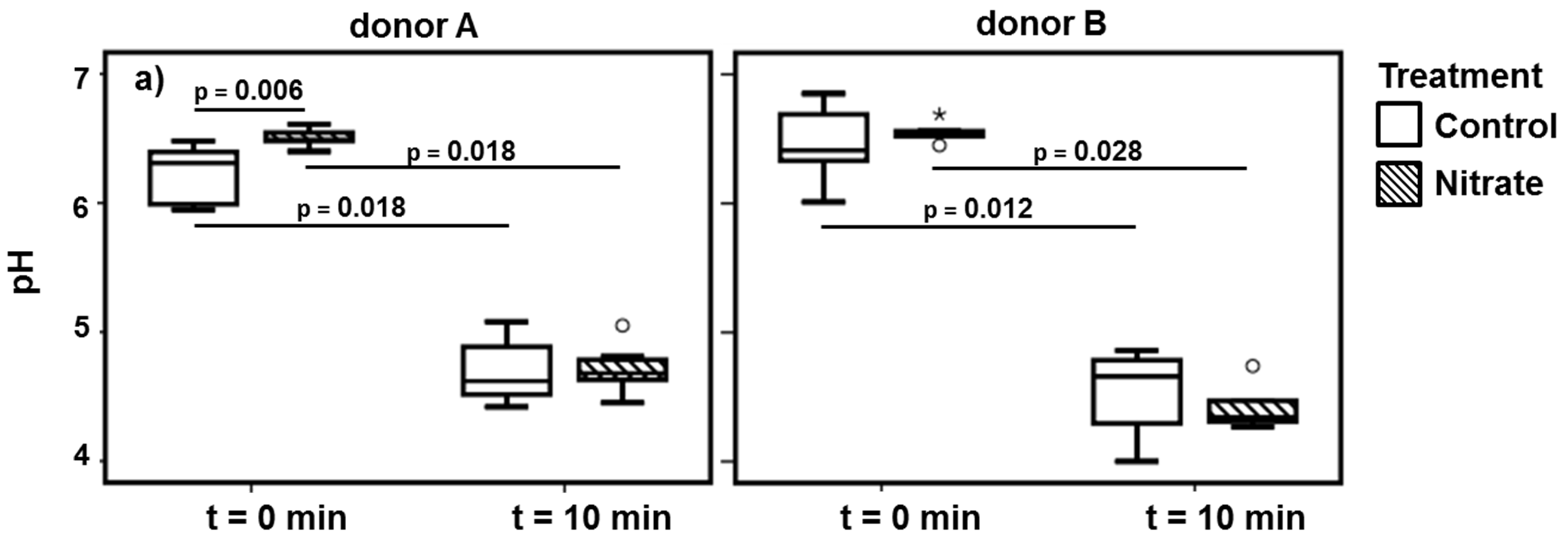

Supplement: Supplementary file 16 — High Resolution (TIF 273 kb) [file 248_2016_775_MOESM9_ESM.tif]

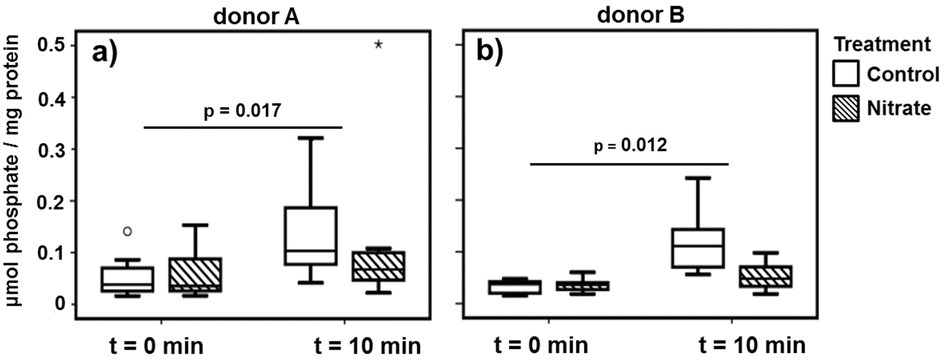

Supplement: Supplementary file 17 — The concentration of phosphate at the start and end point of the sucrose metabolism assay. The concentration of phosphate was measured at the baseline (t = 0 min) and ten min (t = 10 min) after the addition of sucrose to the cell pellets. The significance (p < 0.05) of the difference in phosphate concentration between the time points of the same treatment was tested using the Wilcoxon Signed Ranks Test. The boxes represent the median and interquartile range (IQR), outliers more than 1.5× IQR are depicted by ○, and more than 3× IQR by ★ (GIF 25 kb) [file 248_2016_775_Fig15_ESM.gif]

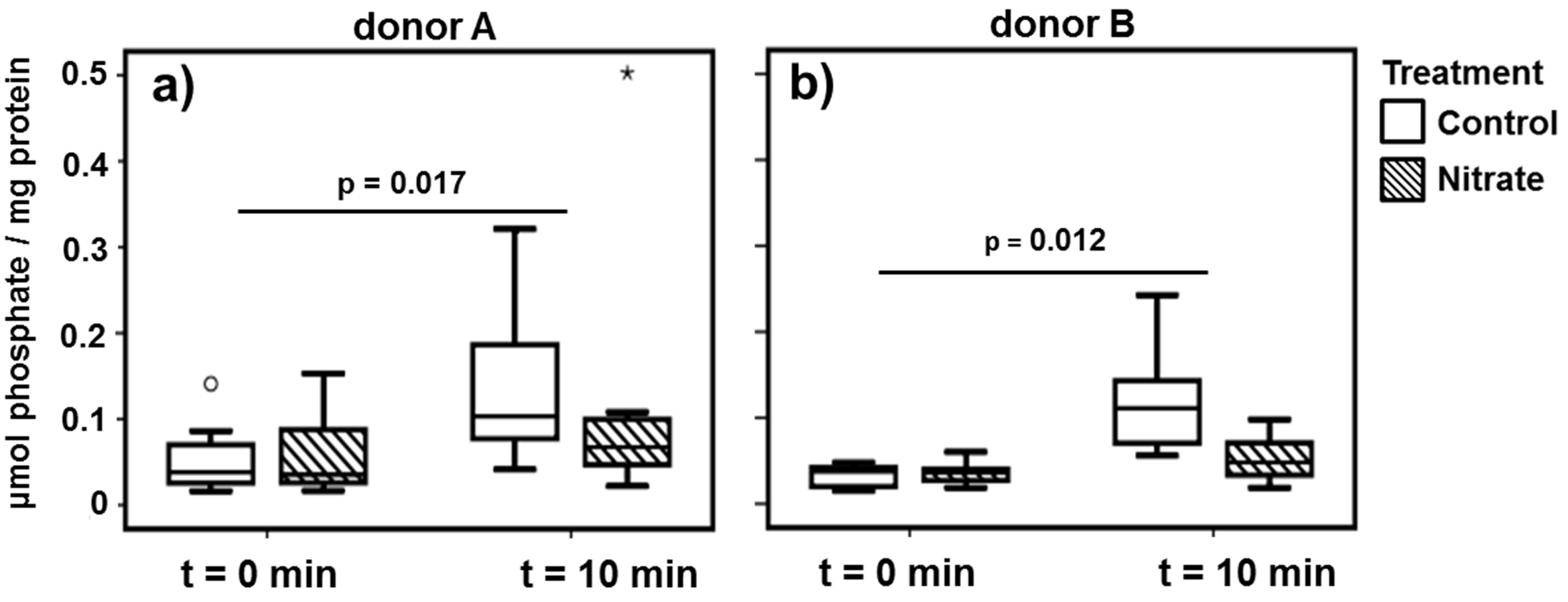

Supplement: Supplementary file 18 — High Resolution (TIF 315 kb) [file 248_2016_775_MOESM10_ESM.tif]
